# How paired PSII–LHCII supercomplexes mediate the stacking of plant thylakoid membranes unveiled by structural mass-spectrometry

**DOI:** 10.1038/s41467-020-15184-1

**Published:** 2020-03-13

**Authors:** Pascal Albanese, Sem Tamara, Guido Saracco, Richard A. Scheltema, Cristina Pagliano

**Affiliations:** 1 Applied Science and Technology Department–BioSolar Lab, Politecnico di Torino, Environment Park, Via Livorno 60, 10144 Torino, Italy; 2 Biomolecular Mass Spectrometry and Proteomics, Bijvoet Center for Biomolecular Research and Utrecht Institute for Pharmaceutical Sciences, University of Utrecht, Padualaan 8, 3584 CH Utrecht, The Netherlands; 3 Netherlands Proteomics Centre, Padualaan 8, 3584 CH Utrecht, The Netherlands

**Keywords:** Proteomics, Mass spectrometry, Light responses, Photosystem II

## Abstract

*Grana* are a characteristic feature of higher plants’ thylakoid membranes, consisting of stacks of appressed membranes enriched in Photosystem II (PSII) and associated light-harvesting complex II (LHCII) proteins, together forming the PSII-LHCII supercomplex. *Grana* stacks undergo light-dependent structural changes, mainly by reorganizing the supramolecular structure of PSII-LHCII supercomplexes. LHCII is vital for *grana* formation, in which also PSII-LHCII supercomplexes are involved. By combining top-down and crosslinking mass spectrometry we uncover the spatial organization of paired PSII-LHCII supercomplexes within thylakoid membranes. The resulting model highlights a basic molecular mechanism whereby plants maintain *grana* stacking at changing light conditions. This mechanism relies on interactions between stroma-exposed N-terminal loops of LHCII trimers and Lhcb4 subunits facing each other in adjacent membranes. The combination of light-dependent LHCII N-terminal trimming and extensive N-terminal α-acetylation likely affects interactions between pairs of PSII-LHCII supercomplexes across the stromal gap, ultimately mediating membrane folding in *grana* stacks.

## Abstract (web summary)

The supramolecular organization of PSII-LHCII supercomplexes determines the plant thylakoid structure. Here, via structural mass spectrometry, Albanese *et al*. show how stroma-exposed N-termini of LHCII subunits, interacting with each other in adjacent membranes, can mediate membrane folding in *grana* stacks.

## Introduction

Biological processes are fundamentally driven by an intricate network of interacting macromolecular protein complexes with large and dynamic structures. These properties make it challenging to resolve structures at atomic detail with classical structural biology approaches, such as crystallography and cryo-electron microscopy (cryo-EM), especially when the complex is buried in a membrane^1^. A multi-subunit pigment–protein complex embedded in the thylakoid membranes of all oxygenic photosynthetic organisms is Photosystem II (PSII). This is an enzyme crucial for life on Earth as, over the past three billion years, powered by solar energy, it  has catalysed the oxidation of water, thus creating the oxygenic atmosphere that sustains all aerobic forms of life^2^. The core complex of PSII is composed of a large number of intrinsic subunits and few extrinsic polypeptides (i.e. PsbO, PsbP, PsbQ and PsbR). Among the membrane proteins there are the large reaction centre D1 and D2 subunits and inner antenna proteins CP43 and CP47, which are accompanied by several subunits with low molecular mass (<10 kDa, e.g. PsbF, PsbK, PsbH, PsbT, etc.), accounting for more than half of the entire complex. The structural organization of the PSII catalytic core has been fundamentally conserved throughout the evolution of photosynthetic organisms from cyanobacteria to higher plants^3^, and its molecular organization has been detailed in previous studies^4,5^. The plant PSII core is serviced by peripheral antenna proteins forming the light-harvesting complex II (LHCII). This antenna system has been prone to evolutionary diversification, producing a wide range of species-specific LHCII isoforms encoded by multiple genes while maintaining a strictly conserved fold and structural organization^6,7^. Different types and numbers of LHCII proteins bind to the PSII core to form PSII–LHCII supercomplexes (PSII–LHCIIsc), whose dynamic remodelling allows plants to adapt to ever-changing environmental light conditions^8,9^.

Plant PSII–LHCIIsc are composed of a PSII dimeric core (C_2_) with two strongly bound (S_2_) LHCII trimers, which are heterotrimers formed by the Lhcb1 and Lhcb2 subunits, and up to two additional moderately bound (M_1–2_) LHCII trimers, containing also the Lhcb3 protein^10,11^. The binding of LHCII trimers to the PSII core relies on three monomeric LHCII subunits, Lhcb4, Lhcb5 and Lhcb6. Lhcb5 acts as the linker exclusively for the S-trimer, Lhcb6 exclusively for the M-trimer and Lhcb4 connects to both trimers^10^. The predominantly occurring PSII–LHCIIsc are of type C_2_S_2_M_2_, C_2_S_2_M and C_2_S_2_^12^, whose relative abundances in the thylakoid membranes depend on the light intensity^9,13^. Due to the intrinsically dynamic arrangement of the outer antenna system it was only recently possible, with the emergence of single particle cryo-EM^14^, to resolve the structure of plant PSII–LHCIIsc at near-atomic detail^15–17^. From these high-resolution structures, Lhcb3 was clearly assigned and exclusively localized within the M-trimer, as the monomer in contact with the Lhcb4 and Lhcb6 subunits^16^. However, not everything could be resolved, as e.g. Lhcb2 could not be differentiated from Lhcb1. Also the atomic structures available for isolated LHCII trimers do not allow discrimination between these two Lhcb proteins^18–20^, as they show high sequence similarity and differentiate mostly at their N-terminus^21^, a feature which is missing in the available high-resolution structures. Their discrimination is difficult even by biochemical methods, although a recent mass spectrometry study performed on preparations of LHCII trimers with different configurations revealed that the M-trimer is enriched in Lhcb1, while Lhcb2 is almost absent in this trimer compared with S-trimers^22^. This result is in accordance with evidence obtained with biochemical studies on isolated PSII–LHCIIsc with different organizations supporting that Lhcb2 is a specific component of the S-trimer, whereas Lhcb1 is present in both S- and M-trimers^12^. Taken together, all these structural and biochemical evidences suggest that: (1) Lhcb3 is present in one copy per M-trimer, likely with mostly two Lhcb1 subunits as partners; (2) Lhcb2 is likely a component only of the S-trimer. As a result, so far little is known about the exact composition and localization of Lhcb1 and Lhcb2 mostly within the S-trimers present in the PSII–LHCIIsc.

The relative flat stromal surface of PSII–LHCIIsc allows it to be accommodated in the tightly stacked region of the thylakoid membranes called *grana*, where the distance between neighbouring membranes is within 2–3.6 nm^10,23^. In plants, the dynamic control of *grana* stacking is crucial for photosynthetic adaptation to light cues^24,25^ and, under variable irradiances, depends on the reversible macro-reorganization of PSII–LHCIIsc^24^. Besides its seminal importance, stacking of *grana* is a topic not yet fully understood^26^. It is thought to be mainly driven by adhesion of LHCII trimers in adjacent membranes^26^, driven by non-specific electrostatic interactions of Lhcb stroma-exposed N-terminal loops^20,27^. Experimental evidences also suggest the involvement of the PSII–LHCIIsc in the stacking of *grana* membranes. Indeed, contacts between PSII–LHCIIsc located in adjacent thylakoid membranes, mediated by the stromal surfaces of both LHCIIs and PSII cores, have been detected in vivo^23^. In addition, unidentified physical connections were observed in the cryo-EM map at 14 Å resolution of paired (C_2_S_2_M)×2 supercomplexes interacting on their stromal side, which were tentatively assigned as mutual interactions of two long N-terminal loops of Lhcb4 spanning the stromal gap^28^. So far the structural determination at high-resolution of these stromal protein–protein interactions by classical structural methods has suffered from multiple limitations: the dynamic nature of the large paired PSII–LHCIIsc assembly (over 2 MDa), the heterogeneity of the LHCII subunits, and the high flexibility of their stroma-exposed N-terminal loops. In addition, N-terminal processing and post-translational modifications (PTMs) such as phosphorylation and acetylation, either on lysine residues or on free termini, occur in the majority of LHCII N-terminal loops^29,30^. For instance, reversible phosphorylation^31^ and lysine acetylation^32^ on Lhcb2 N-terminal loops are central for functional LHCII redistribution during state transitions from PSII, located in *grana* stacks, to Photosystem I (PSI), confined in single-layered thylakoid domains (i.e. *stroma lamellae*). Conversely, permanent N-terminal α-acetylation is known to stabilize proteins and mediate protein–protein interactions^33^, potentially further impacting stromal interactions of paired PSII–LHCIIsc and consequently thylakoid macro-organization.

Considering the complexity of PSII–LHCIIsc, both in terms of light-driven structural dynamics and heterogeneous composition of LHCII, we combined top-down mass spectrometry (TD-MS) and crosslinking mass spectrometry (XL-MS) to resolve so far hidden structural details of paired PSII–LHCIIsc. In this study, we used paired supercomplexes isolated from stacked thylakoid membranes of pea plants grown at three light intensities, ranging from limiting to excessive light. This set allows detection of the stable structural features common to the different conditions and potentially involved in maintaining a basic degree of *grana* stacking. TD-MS is a method capable of identifying intact proteins of up to ~100 kDa^34^ with high throughput and characterizing proteins below ~30 kDa by uncovering mature protein sequences, thus potentially disclosing unknown variants, and PTMs^35^. This approach is particularly suitable for this study as the genome of *P. sativum* (pea) is not fully sequenced. For the XL-MS data analysis, intimate knowledge of the protein sequences is required. Considering the lack of structural features for the flexible stroma-exposed portions of the PSII–LHCIIsc in the high-resolution structure available^16^, and the high homology sequence displayed by the LHCII proteins^21^, the preliminary TD-MS analysis was instrumental for further XL-MS dataset mining and integration. XL-MS, which uses small chemical crosslinkers, has demonstrated considerable potential in gaining structural insights at intermediate resolution on large protein assemblies^36–39^ and even in complex matrixes on a proteome-wide scale^40–42^. This approach is particularly suited for this study as the structure of paired PSII–LHCIIsc from plants is available only at intermediate resolution^28^. We applied TD-MS to profile LHCII isoforms and their proteoforms (i.e. different forms of a protein arising from a given gene with a variety of sequence variants and PTMs). The structures of the most abundant proteoforms, complete with N-terminal stroma-exposed regions, were modelled and fitted into the cryo-EM density map of the (C_2_S_2_M)×2 supercomplex^28^, which represents the most abundant PSII–LHCIIsc common to all three light conditions^9^. To investigate their structural interactions, we treated paired PSII–LHCIIsc isolated from the three light conditions with two complementary chemical crosslinkers, targeting different residues and producing complementary sets of distance restraints^43,44^. Detected crosslinks were used to uncover a tight and specific network of N-terminal loops interacting within the stromal gap and to localize Lhcb2 within the S-trimer. We defined specific sites of interaction between the N-terminal loops of either Lhcb1 or Lhcb2 with PSII core proteins (D1 and CP43 or D1 and PsbH, respectively), potentially acting as hubs to control PSII–LHCIIsc structural dynamics. In addition, we found mutual interactions between N-terminal loops either of Lhcb1 or Lhcb4.2 subunits of adjacent supercomplexes. The two Lhcb4.2 subunits were found to be tightly interacting in a position close to the stromal connecting density defined as the “knot” in the (C_2_S_2_M)×2 cryo-EM map^28^, finally providing an identity to this density. Most interactions detected in vitro on the isolated paired supercomplexes were furthermore supported by XL-MS results obtained in situ on the corresponding thylakoid membranes. These findings represent the first biochemical evidence that mutual interactions between either LHCII trimers or Lhcb4.2 subunits occur between PSII–LHCIIsc facing each other in adjacent thylakoid membranes, suggesting their direct involvement in mediating *grana* stacking.

## Results

### The structure of paired PSII–LHCIIsc

**Fig. 1:**
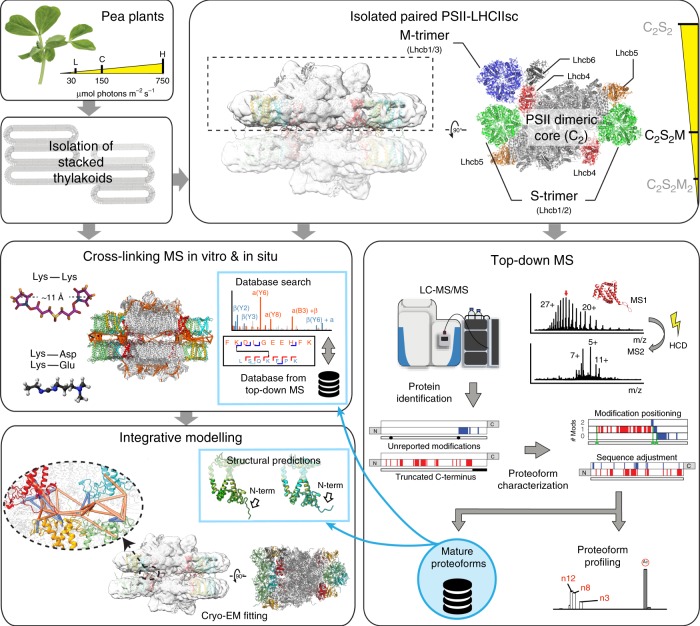
Workflow of the integrated MS-based approach for characterizing the light-driven modulation of paired PSII–LHCIIsc. Isolation of heterogeneous mixtures of PSII–LHCIIsc from pea plants grown at different light intensities (L, low; C, moderate used as control; H, high). PSII–LHCIIsc preparations were used for TD-MS and XL-MS in vitro, performed with DSSO and EDC crosslinkers on two independently isolated PSII–LHCIIsc for each light condition pooled together. DSSO XL-MS in situ was conducted on thylakoid membranes isolated from three independent batches of pea plants grown in moderate (C) light.

As starting material, we used paired PSII–LHCIIsc purified from stacked thylakoid membranes isolated from plants grown at three different light intensities (low, L; moderate used as control, C; and high, H) (Fig. 1). Depending on the light intensity, these samples were enriched in different types of PSII–LHCIIsc, among which the (C_2_S_2_M)×2 was the most abundant and common to all three light conditions^9^ (Fig. 1). To peek through the keyhole of the tight stromal gap between two facing supercomplexes, we combined in-depth TD-MS profiling of intact proteins and detection of protein–protein interactions by XL-MS (Fig. 1). The latter was performed either in vitro on isolated PSII–LHCIIsc or in situ on the starting stacked thylakoid membranes, representing a close-to-native environment.

### The heterogeneity of LHCII unveiled by TD-MS

Given the large degree of heterogeneity expected for individual LHCII subunits, we applied TD-MS to identify proteoforms and estimate relative abundances. For identification we used the medium-/high-resolution workflow previously described (see “Methods” section for details). Of the total detected intensity, approximately 85% could be attributed to identified mass features in any light condition (both in medium-resolution and high-resolution MS1; this does not indicate relative abundance) (Supplementary Data 1). A total of 35 proteins were identified, with 90 different proteoforms, including all the major subunits of the PSII–LHCIIsc. The large subunits CP47 and CP43 and the small subunits PsbH, PsbK, PsbF and PsbT (also referred to as PsbTn^15^) of the PSII core were detected uniquely in the medium-resolution and high-resolution measurement, respectively (Supplementary Fig. 1 and Supplementary Data 1). Less than 10% of the total intensity for all samples was assigned as contaminants, mainly PSI/LHCI subunits and related proteins (Supplementary Fig. 1 and Supplementary Data 1), likely arising from cross-contamination during sample purification^9,45^. Among the detected subunits we accounted for the PSII extrinsic PsbR protein, whose positioning is still debated (i.e. it is absent from high-resolution structures of PSII–LHCIIsc^15–17^), as well as potentially new components of the PSII–LHCIIsc as Psb27 and TL18.3 (here referred to as “PSII-related”) (Supplementary Fig. 1 and Supplementary Data 1). Even though their accumulation appears to be light-dependent (Supplementary Data 1), in agreement with previous findings for PsbR^45^, these soluble proteins are either transiently bound to the PSII in specific light conditions (i.e. Psb27^46^ and TL18.3^47^) or partially lost during PSII–LHCIIsc purification (i.e. PsbR protein^48^). Therefore, from a structural perspective, the study of these proteins requires a dedicated sample preparation to confidently localize their position in the PSII–LHCIIsc architecture.

**Fig. 2:**
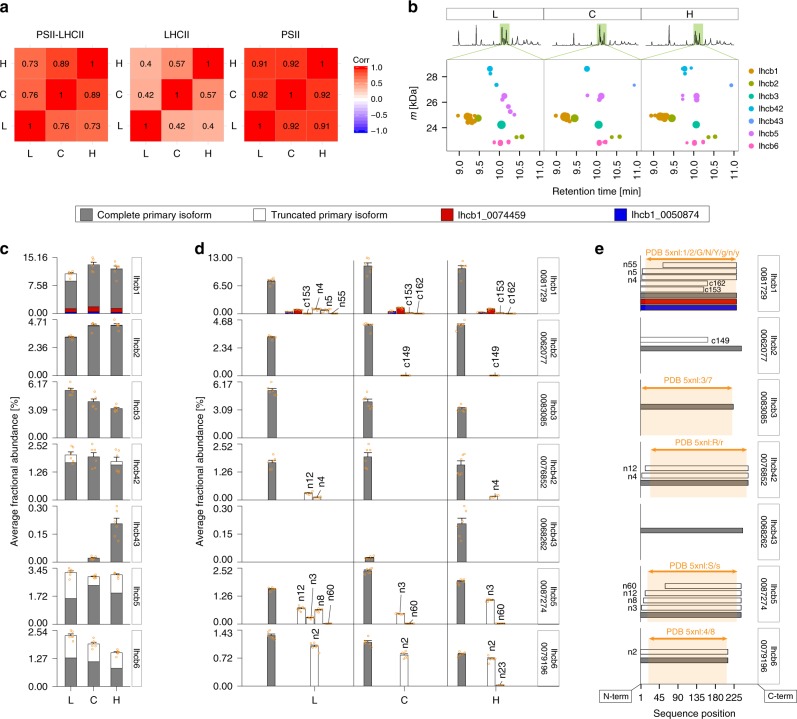
TD-MS profiling of LHCII diversity. **a** Spearman correlation of mass features detected in the PSII–LHCIIsc L, C and H samples for PSII–LHCIIsc (left), LHCII (middle), and PSII (right) proteins. **b** Region of the LC–MS chromatogram corresponding to the elution of LHCII proteoforms displayed as assigned mass features versus retention time (see Supplementary Fig. 1 for overview of all proteins detected in TD-MS). **c** Average abundances of LHCII proteins and **d** detailed average abundances of the distinct isoforms and proteoforms for each LHCII protein, accession number of the primary isoform is reported in the box on the right (see Supplementary Fig. 3 for quantification of distinct proteoforms detected in TD-MS). Error bars represent standard error of the mean abundance for each proteoform. **e** Schematic representation of sequence alignment of LHCII isoforms and proteoforms detected in TD-MS analyses and corresponding sequences resolved in the high-resolution structure of the PSII–LHCII supercomplex from pea plants (PDB: 5xnl, and chains therein; the orange box highlights the portion of protein sequence with resolved structure).

By considering the set of identified PSII–LHCIIsc mass features as a fingerprint for each of the light conditions, it was evident that the major light-dependent variability in proteoform composition occurred within LHCII (Fig. 2a), with distinct patterns emerging when the corresponding monoisotopic masses were plotted against their retention times (Fig. 2b). For each of the LHCII trimer building blocks, Lhcb1, Lhcb2 and Lhcb3, as well as the monomeric Lhcb4 (i.e. Lhcb4.2 and Lhcb4.3), the complete primary isoform was dominant in all light conditions, with only marginal amounts of truncated forms detected (Fig. 2c, d). Conversely, for monomeric Lhcb5 and Lhcb6 the truncated primary isoforms were highly abundant in all light conditions, representing up to 50% of all the detected proteoforms in the L sample (Fig. 2c, d and Supplementary Data 1). Lhcb1 emerged as the most abundant LHCII component, with intensity levels at least two-fold higher than Lhcb2 and Lhcb3 in any light condition (Fig. 2c, d). Such a quantitative assessment is likely correct due to the extremely high sequence homology between these proteins (Supplementary Fig. 2) and structural biases in the detected intensities are therefore not expected. Lhcb1 was present as three distinct isoforms, named according to their transcriptome entries 0081729, 0074459, and 0050874 (Supplementary Data 2). Lhcb1_0081729 accounted for over 80% of all Lhcb1 in any light condition, while the other two isoforms were less abundant. These two isoforms lacked two amino acids at the N-terminus (Ser3 and Ala4), while harbouring four single amino acid substitutions (Supplementary Data 1). Among the remaining identified sequence variants, we found C-terminally truncated forms, two of Lhcb1, cleaved at Asp153 and Asp162, and one of Lhcb2, cleaved at Asp149 (Fig. 2d, e), showing a light-dependent accumulation at increasing light intensities (Supplementary Data 1). Overall, the majority of LHCII proteoforms, with exception of Lhcb2, Lhcb3 and Lhcb4.3, were detected as both complete and truncated forms, primarily with clipping of a few amino acids from the N-terminus (Fig. 2d, e). All the N-terminally truncated forms (with exception of Lhcb6) lacked acetylation on the N-terminal domain, which, conversely, was observed for the majority of the complete forms of Lhcb1, Lhcb2, Lhcb4.2, Lhcb4.3 and Lhcb5 (Supplementary Fig. 3) and the PSII core proteins D1, D2, CP43, PsbF and PsbT (Supplementary Data 1). Among the complete forms of the LHCII proteins, only Lhcb3 and Lhcb6 were primarily observed in non-acetylated state (i.e. unmodified, Supplementary Fig. 3).

The light-dependent modulation of the PSII–LHCIIsc architecture is mainly driven by variations in the amount of the M-trimer subunit Lhcb3, with its specific linker Lhcb6, and of Lhcb4.3 (Fig. 2c). Interestingly, Lhcb4.3 compared to Lhcb4.2 lacks the ~10 amino acids at the C-terminus essential for binding the M-trimer^45^. Indeed, the approximate 50% reduction of Lhcb3 and Lhcb6 in conjunction with an over ten-fold increase of Lhcb4.3 observed in H compared to L, indicate the detachment of M-trimers in high light leading to PSII–LHCIIsc with reduced antennae^9^. Notably, Lhcb2 showed the same light-dependent accumulation trend as Lhcb1 at nearly one-third of the abundance (Fig. 2c). This observation confirms that Lhcb2 is not part of the M-trimer^12^, as otherwise in H light this subunit would decrease in the same fashion as Lhcb3 does. It is also likely that Lhcb2 is present in one copy per S-trimer. We base this on our experimental setup, where the samples were loaded based on the same chlorophyll content and the amount of PSII core proteins and Lhcb1 and Lhcb2 is rather constant in the three light conditions^9^. In fact, if more than one copy of Lhcb2 were present per S-trimer, the Lhcb2:Lhcb1 ratio would increase in H, since in this light condition most of LHCII trimers are of S-type^9^, and in this case we would expect to see, based on MS intensity, a Lhcb2:Lhcb1 ratio of 2:1 instead of the experimentally observed 1:2 shown in Fig. 2c. Even though the abundance comparison provides a rough estimate, it supports the 1:2 stoichiometry between Lhcb2 and Lhcb1, which was further confirmed by absolute quantification at peptide level (Supplementary Fig. 4 and Supplementary Note 1). From the TD-MS we were additionally able to determine most of the remaining PSII–LHCIIsc mature sequences (Fig. 2b, c), whose stroma-exposed N-terminal loops are largely missing in the currently available high-resolution structures^15–17^ (Fig. 2e). These portions are the most diversified (Supplementary Fig. 2b), thus the knowledge of their sequence is determinant for the discrimination of the different LHCII isoforms. As these domains are thought to play a key role in PSII–LHCIIsc structural and functional pairing^23,28^, we continued our investigation with an integrative structural biology approach.

### Interactions between paired PSII–LHCIIsc captured by XL-MS

**Fig. 3:**
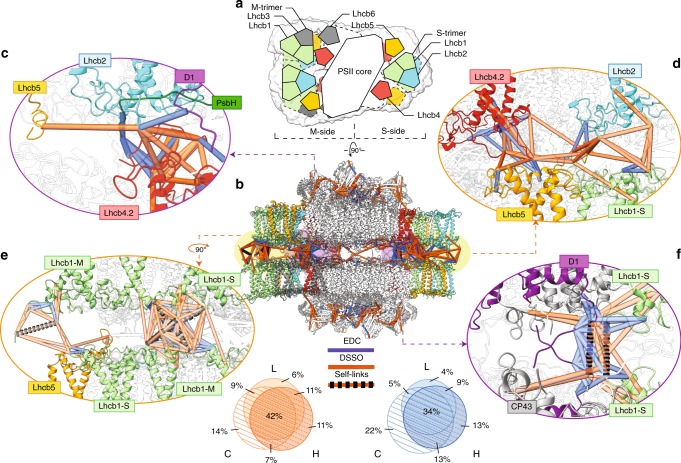
Mapping of crosslinks in the paired PSII–LHCIIsc predicted structural model. **a** Schematic top-view of the (C_2_S_2_M)×2 fitted in the cryo-EM map EMD-3825, showing the overall arrangement of the PSII–LHCIIsc subunits in the predicted models 1–3 (M-side and S-side are indicated). **b** Side-view with mapped crosslinks within the distance cut-off of 17 Å for EDC (blue lines) and 33 Å for DSSO (orange lines), and DSSO self-links (orange-black dashed lines). Venn diagrams showing the overlap between datasets of PSII–LHCIIsc L, C and H samples; only crosslinks present in at least two out of three samples were considered. The enlarged views highlight the crosslinks involving Lhcb2 (**c**, **d**) and Lhcb1 (**e**, **f**) at the periphery of the supercomplex (**d**, **e**) and close to the PSII core (**c**, **f**). Subunits are coloured as follows: Lhcb1 in light green; Lhcb2 in cyan; Lhcb4.2 in red; Lhcb5 in yellow; D1 in purple, PsbH in green and CP43 in grey. Subunits not involved in crosslinks are left transparent.

To uncover details on how the PSII–LHCIIsc structurally interact across the stromal gap, we applied XL-MS using two complementary crosslinking reagents (disuccinimidyl sulfoxide, DSSO; and 1-ethyl-3-(3-dimethylaminopropyl) carbodiimide, EDC) to paired PSII–LHCIIsc preparations representative of L, C and H plants. These preparations are heterogeneous mixtures of the three main types of paired PSII–LHCIIsc (C_2_S_2_)×2, (C_2_S_2_M)×2 and (C_2_S_2_M_2_)×2, whose relative abundances depend on the light intensity^9^. Among them, we focused our structural investigations on the (C_2_S_2_M)×2, representing roughly half of all paired PSII–LHCIIsc in any light condition^9^. Identified crosslinks present in at least two out of three light conditions were mapped on the (C_2_S_2_M)×2 model derived from the cryo-EM density map from pea plants^28^ (Fig. 1). The crosslinking reactions, performed in solution under mild conditions (see “Methods” section for details), preserved paired PSII–LHCIIsc from random aggregation while maintaining their stromal interactions (Supplementary Fig. 5). We found that inclusion of the TD-MS-derived sequence variants into the database search was a key step, as the number of detected crosslinks compared to the non-supplemented available database was almost doubled (from 161 to 304 considering the DSSO dataset for C light, Supplementary Data 2 and 3). In total, we identified 260, 304 and 289 crosslinks with DSSO and 358, 479 and 495 with EDC for the L, C and H samples, respectively (Supplementary Data 3). The overlap between the datasets from the three light conditions was ~42% for DSSO and ~34% for EDC, with 69% and 61% of the crosslinks present in at least two out of three samples, respectively (Fig. 3). These two crosslinkers used in tandem proved to be highly complementary, while showing also a reproducible pattern of subunit linkage between the different illumination conditions (Supplementary Fig. 6). This suggests that the overall structure of the paired PSII–LHCIIsc does not undergo major structural light-driven remodelling. A small amount of PSI/LHCI proteins was found in low abundance in both the TD- and XL-MS datasets (Supplementary Data 1 and 3), but a lack of inter-links with PSII–LHCIIsc proteins (Supplementary Fig. 6) supports that these rather arose from cross-contamination. The new components of PSII–LHCIIsc suggested by the TD-MS results (i.e. TL18.3 and Psb27, Supplementary Fig. 1 and Supplementary Data 1) exhibited a low reproducibility at the XL-MS level (Supplementary Fig. 6 and Supplementary Data 3), likely due to their differential accumulation in the different samples (Supplementary Data 1). Uncovering their positioning in the wider PSII–LHCIIsc structure will require a dedicated sample preparation in future experiments. The amount of intra-protein crosslinks was ~40% (Supplementary Data 3), a lower percentage compared to that commonly found in this type of study. Since most of the PSII–LHCIIsc subunits are densely packed transmembrane proteins, for which the membrane itself provides steric hindrance reducing solvent-accessible residues, this low percentage is likely determined by the reduced access for the crosslinkers to the transmembrane helices. Indeed, no crosslinks were found on the membrane buried parts of the proteins and many of the detected crosslinks involved flexible loops of several PSII–LHCIIsc components. Most of these (35–45% of total crosslinks in every sample) belonged to stroma-exposed LHCII N-terminal loops, which incidentally were not detected when using the non-supplemented protein database (Supplementary Data 3).

Considering the evolutionary diversity and extensive light-dependent conformational variation of the plant LHCII antenna system and, conversely, the high conservation of its PSII core^3^, we validated the DSSO and EDC datasets on the high-resolution structure available for the pea plant PSII core^16^. By mapping the crosslinks detected for this region, we were able to confidently place 66 crosslinks for DSSO and 61 for EDC, 87% and 74% of the total PSII core detected crosslinks, respectively (Supplementary Fig. 7). The remaining set of 13% and 26% crosslinks violating the strict cut-off distance (i.e. >33 Å for DSSO and >17 Å for EDC) can be however largely validated considering an acceptable large cut-off of <35 Å for DSSO^49^ and <30 Å for EDC^50^ (Supplementary Fig. 7). These over-length crosslinks involved mainly PsbO and PsbP, two extrinsic subunits protruding on the lumenal side of the PSII core, suggesting that limited structural rearrangements occur within these domains. Overall, the structural validity of the majority of the PSII core detected crosslinks supports that under the conditions applied also crosslinks involving the peripheral LHCII can be considered significant.

### Structural modelling of LHCII explains stromal interactions

A large number of detected crosslinks involved N-terminal regions. The structural details of the protein interactions they represent are impossible to resolve without access to the N-termini, regions that are missing from the high-resolution structures available so far^15–17^ (e.g. for LHCII see Fig. 2e). To overcome this limitation, the structures of all LHCII proteins along with D1, D2, CP47 and PsbH of the PSII core with unknown N-terminal atomic details were predicted by structural modelling (Supplementary Data 4) using the sequences of their most abundant proteoform uncovered by TD-MS (Supplementary Data 1). Substitution of these predicted subunits into the pea PSII–LHCIIsc high-resolution structure^16^ can be confidently performed considering the restricted changes to the PSII–LHCIIsc structures in diverse plant species^15–17^ and that they were used as templates. These models were subsequently fitted into the cryo-EM structure of the (C_2_S_2_M)×2^28^, significantly increasing the number of validated crosslinks, with e.g. an increase from 143 to 232 in the DSSO dataset for C light (Supplementary Fig. 8). Placement of Lhcb2 within the PSII–LHCIIsc structure was driven by 28 unique inter-protein crosslinks involving Lhcb2 (excluding two inter-links whose sequences overlapped with Lhcb1; Supplementary Data 3) and the assumption of one copy of Lhcb2 per S-trimer as provided by the TD-MS results. We generated nine theoretical models where Lhcb2 substituted one Lhcb1 in any possible position within the S-trimer, either on the M-side or on the S-side of the supercomplex (Supplementary Fig. 9 and Supplementary Data 4). The most probable position of Lhcb2 was determined by combining DSSO and EDC inter-protein crosslinks involving this subunit (Supplementary Data 5) and ranking the models considering the score of the search algorithm and the number of crosslinks involving Lhcb2 validated within the distance threshold (see “Methods” section for details). By ranking the nine theoretical models, we found that the highest-ranking model, used hereafter, placed the Lhcb2 within the S-trimer close to the PSII core on the M-side and peripherally on the S-side of the supercomplex (corresponding to models 1–3 in Supplementary Fig. 9 and Supplementary Data 4). Two clusters of crosslinks defined the interactors of Lhcb2 in the predicted (C_2_S_2_M)×2 (Fig. 3a, b) as: (1) near the PSII core on the M-side, the Lhcb2 N-terminal loop interacts with the PsbH N-terminus and D1′Glu6 residue, in addition to Lhcb4.2 and Lhcb5 (Fig. 3c); and (2) peripherally on the S-side, Lhcb2 interacts with Lhcb1 and Lhcb5 (Fig. 3d).

### Lhcb1 determines PSII–LHCIIsc pairing across the stromal gap

The predicted model for the (C_2_S_2_M)×2 showcased an intricate network of subunits interacting across the stromal gap (Fig. 3b). Indeed, 104 crosslinks for DSSO and 54 for EDC were uniquely attributable to subunits interacting across the stromal gap. We found that Lhcb1 N-terminal loops of facing supercomplexes mutually interact, forming well-defined and intricate clusters. These clusters are localized either on the M-side, where six Lhcb1 proteins are peripherally superimposed (Fig. 3a, e), or on the S-side, where two facing Lhcb1 proteins interact with the PSII core by forming a cluster with the N-terminal loop of D1 (Lhcb1′Lys2 and Lhcb1′Lys7 crosslink either D1′Glu5 or D1′Asp8; Lhcb1′Lys2 crosslinks also D1′Glu10) and CP43 (i.e. inter-link Lhcb1′Lys2-CP43′Lys457) (Fig. 3a, f). Lhcb1 mutual interactions were also supported by self-links, crosslinked peptide pairs involving the same lysine residue (i.e. Lhcb1′Lys2–Lhcb1′Lys2 and Lhcb1′Lys8–Lhcb1′Lys8) in peptides with different missed cleavages, which can only occur if the Lhcb1 interacts with itself across the stromal gap (Fig. 3b–f, Supplementary Fig. 10).

Mutual interactions between distinct copies of Lhcb1 in adjacent supercomplexes were detected in all three light conditions (Supplementary Data 3); conversely, such interactions between Lhcb2 subunits were not observed (Supplementary Data 3). This finding agrees with the distant position of the two Lhcb2 subunits in our structural model (Fig. 3a) and suggests a secondary role for Lhcb2 with respect to Lhcb1 in PSII–LHCIIsc pairing. Furthermore, considering the Lhcb1:Lhcb2 ratio ranging between 2:1 in a C_2_S_2_ and 4:1 in a C_2_S_2_M_2_, as deduced from our structural model placing Lhcb2 uniquely in the S-trimer, and the higher ratio of Lhcb1 over Lhcb2 observed by TD-MS in the PSII–LHCIIsc in any light condition tested (Fig. 2), this finding further suggests an important role for Lhcb1 in maintaining LHCII trimer superimposition in any type of paired PSII–LHCIIsc at changing light conditions.

### Lhcb4 N-terminal loops anchor the paired PSII–LHCIIsc

**Fig. 4:**
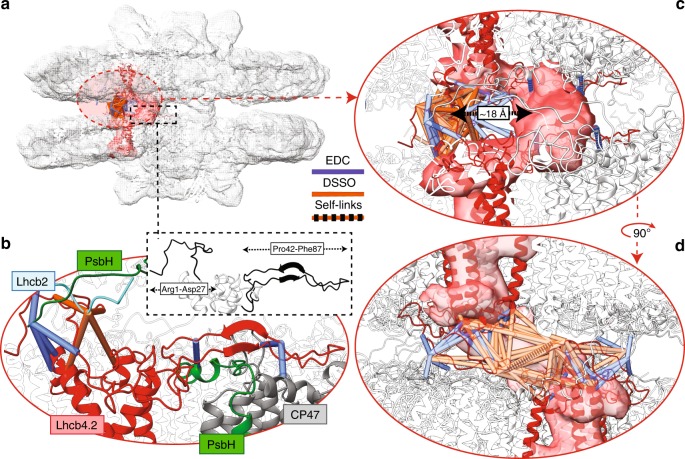
Mapping of Lhcb4.2 crosslinks putatively responsible for the structural anchor of paired PSII–LHCIIsc. **a** Side-view of the (C_2_S_2_M)x2 fitted in the cryo-EM map EMD-3825, with the “knot” connecting density highlighted in red. **b** Enlarged view highlighting the inter-protein crosslinks involving the N-terminus (Arg1–Asp27) and the long hairpin (Pro42–Phe87) of Lhcb4.2. These two regions of the N-terminal loop of the Lhcb4.2 are shown in black in the inset. Side-view **c** and end-view **d** of the putative site of interaction between the flexible N-termini (Arg1–Asp27) of two Lhcb4.2 subunits facing from adjacent supercomplexes are shown, and their ~18 Å displacement from the “knot” density is indicated. Crosslinks within the distance cut-off of 17 Å for EDC (blue lines) and of 33 Å for DSSO (orange lines), and DSSO self-links (orange-black dashed lines) are shown. Subunits are coloured as follows: Lhcb2 in cyan; Lhcb4.2 in red; PsbH in green and CP47 in grey. Subunits not involved in crosslinks are left transparent.

Lhcb4 occupies a pivotal position within the PSII–LHCIIsc, serving as a linker for either S- or M-trimers. Owing to the proximity of two facing Lhcb4 subunits on the M-side of a paired PSII–LHCIIsc (Fig. 3a), these proteins were previously suspected to provide a structural anchor between facing supercomplexes by tying the “knot” connection through the mutual interaction of their long N-terminal loops^28^ (Fig. 4a). We identified this subunit by TD-MS (Supplementary Data 1) and, based on the complete amino acid sequence, detected numerous crosslinks (Fig. 4b–d). Although we detected two isoforms of Lhcb4 (i.e. Lhcb4.2 and Lhcb4.3, Fig. 2b–e), we only placed Lhcb4.2 into our structural model as it is at least tenfold higher in abundance and, importantly, its amount is stable in all light conditions (Fig. 2c, d). Combined, this led to more reproducible crosslinks for Lhcb4.2 than for Lhcb4.3 (Supplementary Data 3). We detected crosslinks involving the stroma-exposed long hairpin (Pro42–Phe87) of Lhcb4.2 and the PSII core proteins CP47 and PsbH (i.e. Lhcb4.2′Glu85-PsbH′Lys24, Lhcb4.2′Asp74-CP47′Lys227 and Lhcb4.2′Asp74-CP47′Lys130) (Fig. 4b and Supplementary Data 3). These crosslinks indicate that at least half of this hairpin runs mostly parallel to the stromal surface, as observed in previous PSII–LHCIIsc structures^15–17^. In these structures, however, the N-terminal domain (Arg1–Asp27) was not resolved. We detected more than 20 crosslinks involving Lhcb4.2 N-terminus (Arg1–Asp27) in all light conditions, among which one was a self-link (here defined as intra-link between neighbouring lysine residues of Lhcb4.2, below the minimum DSSO cut-off distance of ~7 Å) (Supplementary Data 3 and Supplementary Fig. 10). This self-link can unambiguously be assigned to mutual interactions between two Lhcb4.2 subunits (Fig. 4c, d), which act as a structural anchor for the PSII–LHCIIsc pairing across the stromal gap. Their putative interaction site inferred from the cryo-EM structure (i.e. the “knot”) is ~18 Å from the site predicted by our XL-MS data (Fig. 4c). Considering that no constraints were imposed for the structural prediction of Lhcb4.2, it is conceivable that the exact position of the two interacting N-terminal loops is slightly shifted, justifying some degree of mobility that allows their accommodation within the “knot” density.

### In situ XL-MS validates PSII–LHCIIsc pairing in thylakoids

**Fig. 5:**
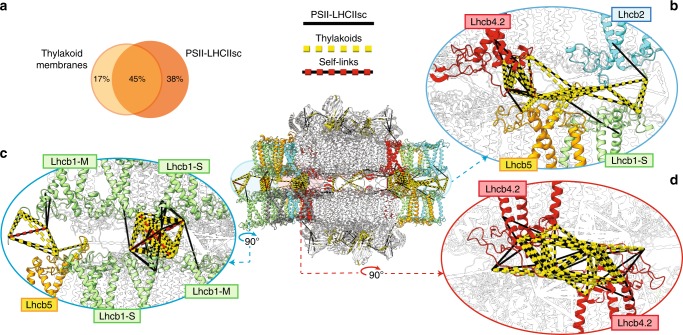
Detection of paired PSII–LHCIIsc in stacked thylakoid membranes by in situ XL-MS with DSSO. **a** Side-view of the (C_2_S_2_M)×2 with mapped crosslinks found in at least two out of three thylakoid samples and two out of three PSII–LHCIIsc samples, together with the overlap of the two datasets. Enlarged views matching Figs. 3d, e and 4d are shown to highlight the positioning of Lhcb2 (**b**) and the occurrence of Lhcb1 and Lhcb4.2 mutual interactions (**c**, **d)** in the thylakoid membranes. Crosslinks within the distance cut-off of 33 Å for DSSO either for isolated PSII–LHCIIsc (black lines) or thylakoid membranes (yellow dashed lines), and DSSO self-links (red dashed lines) are shown. Subunits not involved in crosslinks are left transparent.

Whether the structures of protein complexes seized from their cellular milieu represent their native conformation is still largely debated. To verify our results in a close-to-native state, we applied the DSSO XL-MS workflow to thylakoid membranes isolated in stacked conformation from plants grown in moderate light intensity (C), prior to solubilization and PSII–LHCIIsc purification. Owing to the considerable amounts of PSII–LHCIIsc embedded in the thylakoids, we were able to detect 296, 302 and 293 crosslinks attributable to PSII–LHCIIsc subunits in each of the three independent replicates. Of these, only 64, 70 and 67 were unique for each replicate, resulting in an overlap of ~77% of crosslinks common to at least two replicates (Supplementary Data 6). Notably, the crosslinking pattern obtained in situ showed similar subunit linkages compared to in vitro (Supplementary Fig. 6), with most of the crosslinks detected in situ in two out of three replicates also detected in vitro in PSII–LHCIIsc in at least two out of three light conditions (Fig. 5a). These results suggest that the XL-MS predicted model for the paired PSII–LHCIIsc can be regarded as structurally valid. The coherent positioning of Lhcb2 in the predicted structural model was supported by a reproducible network of crosslinks also found in situ between Lhcb1, Lhcb2, Lhcb4.2 and Lhcb5 (Fig. 5b). PSII–LHCIIsc structural pairing across the stromal gap was also unambiguously supported by the occurrence of self-links of Lhcb1 (i.e. Lhcb1′Lys8–Lhcb1′Lys8) (Fig. 5c) and Lhcb4.2 (i.e. Lhcb4.2′Lys10–Lhcb4.2′Lys10 and Lhcb4.2′Lys8–Lhcb4.2′Lys10) (Fig. 5d) (for corresponding spectra see Supplementary Fig. 11). The coherence between in situ and in vitro DSSO XL-MS results supports that LHCII of PSII–LHCIIsc facing from adjacent membranes of stacked thylakoids structurally interact within the native environment through mutual interactions of Lhcb4.2 and Lhcb1 N-terminal loops.

### Extensive N-terminal acetylation occurs in the stromal gap

**Fig. 6:**
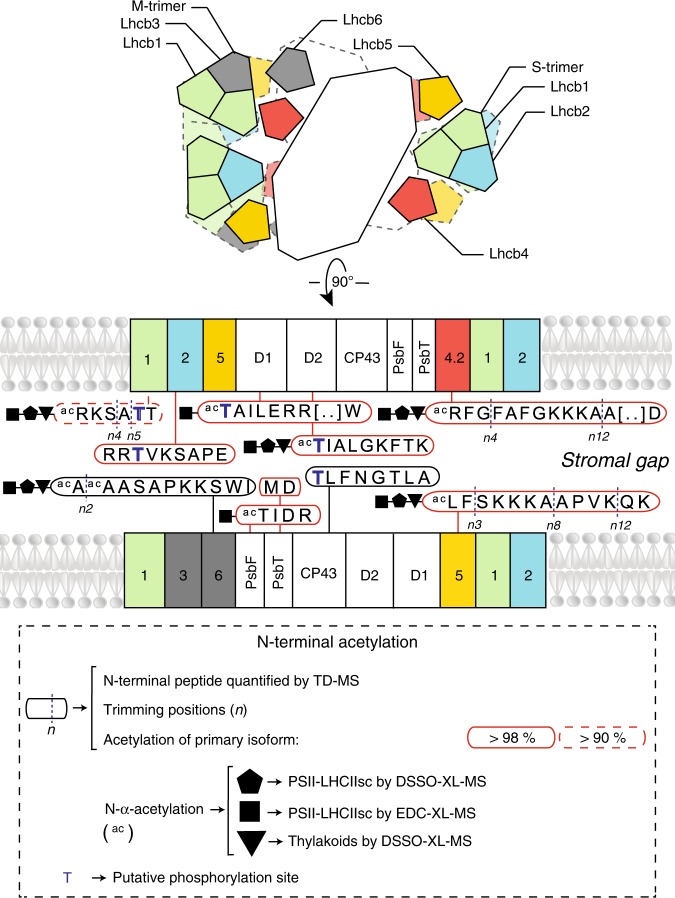
Map of acetylated N-terminal domains of PSII–LHCIIsc subunits spanning the stromal gap. Schematic top- and side-view of the (C_2_S_2_M)×2, highlighting the acetylated N-terminal domains of the proteins spanning across the stromal gap detected and quantified by TD-MS (red box indicates acetylation rate of the complete primary isoform in any light condition, above 98% solid line; above 90% dashed line). Trimming position(s) and putative phosphorylation sites (based on homologous phosphosites previously detected in other plants^29^) are indicated. N-α-acetylation (ac) detected in crosslinked peptides is shown. N-α-acetylation detected by XL-MS in vitro on isolated PSII–LHCIIsc (treated with DSSO, pentagon; treated with EDC, square) and in situ on the thylakoid membranes (treated with DSSO; triangle) is shown.

Both thylakoid stacking and PSII–LHCIIsc pairing depend on the presence of cations^28,51^. Conversely, thylakoid unstacking is triggered by reversible phosphorylation of stroma-exposed N-terminal loops, introducing negative charges on membrane surfaces^26^. Here, we found that irrespective of the light condition, acetylation is a widespread PTM on the stroma-exposed N-terminal loops of many of the PSII–LHCIIsc proteins in stacked thylakoid membranes, which can be reproducibly detected by both TD-MS and XL-MS (Fig. 6 and Supplementary Data 1). Indeed, in any light condition most of the primary non-truncated isoforms of Lhcb1, Lhcb2, Lhcb4.2 and Lhcb5, as well as the PSII core proteins D1, D2, PsbF and PsbT were found to be acetylated in one of the first 20 amino acids by TD-MS (Fig. 6, Supplementary Fig. 3 and Supplementary Data 1). Characterization of PTMs on crosslinked peptides involving stroma-exposed PSII–LHCIIsc terminal loops, either in vivo or in situ, pinpointed acetylation predominantly as N-α-acetylation, which occurred only on proteins with a complete N-terminus (with exception of an acetylated truncated Lhcb6) (Fig. 6 and Supplementary Fig. 3). The lack of evidence for N-α-acetylation in Lhcb2 crosslinked peptides (Supplementary Data 3 and 6), despite the detection of this PTM at its N-terminal domain (Fig. 6), suggests the occurrence of a lysine acetylation in this region (i.e. likely on Lys5^30^), whose interplay with phosphorylation of Lhcb2-Thr3 is required to trigger the state transitions^32^. Notably, our data suggest that stable N-α-acetylation and reversible phosphorylation at the N-terminal domain might play concerted roles. This is based on the potential occurrence of both PTMs in D1 and D2 on the first Thr (Fig. 6), as previously reported^52^, and on Lhcb1 and Lhcb4.2 (i.e. the main Lhcbs involved in PSII–LHCIIsc pairing), where N-α-acetylation occurs on the first Arg while the putative phospho-sites are localized elsewhere^29^ (Fig. 6).

## Discussion

The peculiar bipartite structure of plant thylakoid membranes, consisting of *grana* stacks and helically wound *stroma lamellae*, undergoes light-dependent dynamic structural changes predominantly by remodelling the supramolecular organization of PSII–LHCIIsc within the *grana*. Although PSII–LHCIIsc can be isolated in paired conformation from stacked thylakoid membranes^16,28^, the available cryo-EM structures suffer from extensive ensemble averaging, resulting in loss of structural details of sub-stoichiometric subunits and proteoforms with flexible domains, such as the heterogeneous LHCIIs, which are thought to play a role in *grana* stacking^20,23,27^.

In this work, we demonstrated how the integration of in-depth TD-MS profiling of intact proteoforms is beneficial for achieving comprehensive XL-MS analyses performed concomitantly in vitro on isolated supercomplexes and in situ on thylakoid membranes. Demonstrating the feasibility of such an integrated approach, this work paves the way for future disentanglement of the structural dynamics of plant PSII–LHCIIsc in response to light cues. This task might be achieved by performing an accurate quantitative XL-MS analysis (e.g. through TMT-labeling) once cryo-EM structures at intermediate resolution become available for PSII–LHCIIsc isolated from plants grown under different irradiances. In addition, also the potentially novel interactors of the PSII–LHCIIsc uncovered in this study (i.e. the lumenal TL18.3 and Psb27 proteins, Supplementary Fig. 1 and Supplementary Data 1) would benefit from such an integrated structural study.

Combining results from TD-MS, XL-MS, and integrative modelling, we were able to construct and validate a structural model of (C_2_S_2_M)×2 with one copy of Lhcb2 per S-trimer at a ratio 1:2 with Lhcb1 (Fig. 3). So far cryo-EM produced high-resolution structures of plant PSII–LHCIIsc that, due to high sequence homology between Lhcb1 and Lhcb2, were unable to reveal the exact composition of LHCII trimers in terms of Lhcb1 and Lhcb2 stoichiometries as well as the precise localization of these two isoforms therein^15–17^; our hybrid MS approach did succeed in this challenging task. So far the Lhcb2 structure has only been disclosed in the mobile LHCII trimer involved in binding PSI during state transitions^53^. Intriguingly, this trimer showed the same subunit composition of the S-trimer determined in this work. The Lhcb1 was the most abundant Lhcb detected (Fig. 2c), and mutual interactions of Lhcb1 N-terminal loops across the stromal gap were found to bridge facing PSII–LHCIIsc either in vitro (Fig. 3) or in situ (Fig. 5). Similar mutual interactions were detected between N-terminal loops of Lhcb4.2 subunits (Figs. 4 and 5). These results provide clear biochemical evidence for the role of Lhcb1 N-terminal loops to enforce thylakoid stacking supporting the so-called “Velcro effect”^20,23^. This effect was hypothesized to be driven by interactions of positively charged amino acids at the N-terminus with the negatively charged stromal surface of LHCII, whose mutual interactions across the stromal gap are mediated by cations^27^. Intriguingly, both Lhcb1 and Lhcb4.2 showed extensive N-terminal truncation in the L sample, with removal of the first positively charged amino acids (Figs. 2d, e and  6). Similarly, a positively charged tail made of the Lys3, Lys4 and Lys5 residues was missing in the *n8* and *n12* truncated Lhcb5, which was found most abundant in the L sample. Conversely, this positively charged tail was present in the *n3* truncated proteoform, which was found enriched in the C and H samples (Fig. 2d, e and  6). The presence in low light of truncated forms of Lhcb1, Lhcb4.2 and Lhcb5 lacking either positive N-terminal tails or N-α-acetylation (Fig. 6 and Supplementary Data 1), the latter known to stabilize proteins^54^, might determine an overall imbalance of surface charges. Furthermore, the accumulation of destabilizing truncated Lhcb1 forms in low light indicates their preferential localization within M-trimers, whose selective undocking from PSII–LHCIIsc is advantageous for plants to cope with excessive irradiation. Accordingly, we found limited amounts of N-α-acetylation on Lhcb6 and absence of this PTM on Lhcb3 (Fig. 6 and Supplementary Fig. 3), leaving unshielded the N-termini of these two M-trimer-related proteins, which are abundant in low light (Fig. 2c). This charge-modulated interaction between specific LHCII N-terminal loops may serve to finely tune M-trimers docking to the PSII core and PSII–LHCIIsc pairing across the stromal gap. This is further exacerbated when negative charges are introduced through extensive Thr phosphorylation of stroma-exposed residues^29^, which are excluded from N-terminal truncations (Fig. 6). Light-dependent phosphorylation of LHCII and PSII components appears to initiate most of the regulatory mechanisms that lead to thylakoid structural changes in response to environmental light variations^26,55^. Since we used dark-adapted samples, extensive phosphorylation was neither expected^29^ nor observed. However, the N-terminal tail networks of Lhcb1 and Lhcb4.2 uncovered in this study, locking the supercomplex in place (Fig. 3), might facilitate the access through the narrow stromal gap^10,23^ for the STN7 and STN8 kinases, which are responsible for the light-dependent phosphorylation of the stroma-exposed N-terminal loops of LHCII and PSII core proteins, respectively^56–58^ (Fig. 6). Indeed, two functional “hubs” appear in our (C_2_S_2_M)×2 structural model, where (1) Lhcb4.2 and Lhcb2 interact closely with D1 and PsbH on the M-side (Fig. 3c) and (2) Lhcb1 forms a tight network with D1 and CP43 on the S-side (Fig. 3f). As D1 is buried in the bulk of the PSII core and its phosphorylation is crucial for the regulation of the whole photosynthetic process^59^, these hubs might play a key role in triggering the structural reorganization of PSII–LHCIIsc in response to light cues.

The results obtained highlight the occurrence of a basic molecular mechanism based on mutual interactions of LHCII trimers and Lhcb4.2 N-terminal loops bridging facing PSII–LHCIIsc across the stromal gap, that together with a widespread light-independent N-α-acetylation of stroma-exposed N-terminal loops ultimately strengthen *grana* stacking at any light condition. In conclusion, the power of TD-MS in disentangling LHCII heterogeneity was indispensable for XL-MS and integrative structural modelling to unravel the structural details of paired PSII–LHCIIsc in vitro and in situ, providing evidence of their involvement in mediating the *grana* stacking in plants.

## Methods

### Isolation of thylakoids and PSII–LHCIIsc purification

*P. sativum* L. plants were grown inside the growth chamber SANYO MLR-351H at 20 °C and 60% humidity for 3 weeks under 8 h daylight^60^ at three different light intensities, 30 (low, L), 150 (moderate used as control, C) and 750 (high, H) µmol photons m^−2^ s^−1^ . The L and C conditions were provided by turning on 3 and 15 fluorescent lamps (FL40SS W/37) in the growth chamber, respectively; H condition was supplied by four LEDs (LXR7-SW50) mounted inside the growth chamber^61^. Stacked thylakoid membranes were isolated at the end of the daily dark phase, using buffers supplemented with divalent cations (Mg^2+^)^62^ to mimic the native chloroplast ionic conditions^63^ and preserve the stacked morphology of the *grana* membranes. After mild solubilization of stacked thylakoid membranes with 50 mM n-dodecyl-α-d-maltoside, purification of paired PSII–LHCIIsc was performed by sucrose gradient ultracentrifugation in the dark with a buffer containing divalent cations (i.e. 5 mM Mg^2+^) at mild acidic pH (5.7) to preserve their macro-organization and functionality^16,28^.

### Top-down sample preparation and LC–MS/MS

A total of 100 µg of PSII–LHCIIsc for each light condition (L, C and H) was buffer exchanged into 10% Formic acid by using 5000 MWCO VIVASPIN centrifuge filters (Vivaproducts Inc., Littleton, USA). The final mixture was then diluted to a final concentration of 1 µg/µL. To avoid light-induced degradation, samples were prepared under dim green light and kept in amber glass thread vials during all consequent steps. Chromatographic separation was performed on a Thermo Scientific Vanquish Flex UHPLC instrument coupled on-line with a MAbPac reversed-phase analytical column (2.1 mm × 50 mm) heated to 80 °C to a Q Exactive HF-X instrument (Thermo Fisher Scientific, Bremen, Germany)^64^. A total of 2–3 µg of material was loaded onto the analytical column and separated over 36 min at a flow rate of 250 µL/min. Gradient elution was performed using mobile phases A (H_2_O/0.1% CH_2_O_2_) and B (C_2_H_3_N/0.1% CH_2_O_2_): 25–60% B ramp-up in 34 min. Each sample run was followed by two cleaning cycles with increasing mobile phase B from 10% to 100% and column equilibration for 10 min with 90% buffer A. The high amount of chlorophylls and carotenoids carried by LHCII proteins can significantly hamper protein detection inside the mass spectrometer regardless of ionization conditions. However, during the reversed-phase liquid chromatography (RP-LC) step, the strong binding of these molecules to the analytical column led to elution of proteins stripped of all pigments simplifying the mass analysis.

LC–MS(/MS) data were collected with the mass spectrometer set to the Intact Protein Mode and trapping gas pressure set to 0.2. During analysis, two methods were used with complementary resolutions in full MS mode, either medium-resolution of 7500 at 200 *Th* or high-resolution of 120,000 at 200 *Th*^65^. Although the medium-resolution allows for improved detection of ions with masses above ~30 kDa, at this resolution the instrument lacks sensitivity for detecting low-mass ions. In contrast, the high-resolution provides accurate mass detection for ions with masses below ~30 kDa but not above. Full MS scans were acquired for the range of 400–2400 *Th* with AGC target set to 3e6. The maximum injection time was set to 16 ms with 1 µscan recorded for the medium-resolution and 250 ms with 5 µscans for the high-resolution scans. All MS/MS scans were recorded with a resolution of 120,000, a maximum injection time of 250 ms, an AGC target of 3e6 and 5 µscans for the three most intense proteoforms in each cycle as determined by the advanced precursor determination algorithm^35^. The ions of interest were mass selected by quadrupole in a 2 *Th* isolation window and collected to an AGC Target of 3e6 ions prior to fragmentation at NCE = 30. Only the single most intense charge state was selected for isolation/fragmentation in dd-MS/MS per deconvoluted peak array with other charge states excluded from the candidate list for an exclusion time of 6 s.

### Top-down data analysis

A custom protein database was derived from the transcriptome of *P. sativum* (p.sativum_csfl_reftransV1 downloaded from https://www.coolseasonfoodlegume.org/organism/Pisum/sativum/reftrans/v1) and supplemented with homologous sequences available for *P. sativum* in UniProtKB/TrEMBL^61^ (version 17.01.2019 containing 1803 sequences). Isotopically resolved or unresolved spectra were deconvoluted, respectively, with either Xtract^66^ or ReSpect (Thermo Fisher Scientific, Bremen, Germany). Automated searches against our database were performed in Thermo Proteome Discoverer (version 2.3.0.522) extended with the ProSightPD nodes for Medium–High (medium-resolution in full MS) and High–High (high-resolution in full MS) experimental workflows. Parameters for the Medium–High method were set as follows. ReSpect: precursor *m/z* tolerance—0.2 *Th*; relative abundance threshold—0%; precursor mass range—3–100 kDa; precursor mass tolerance—30 ppm; charge state range—3–100. Xtract: signal/noise threshold—2; *m/z* range—400–2400 *Th*. Initially, a large precursor tolerance window of 10 kDa was set to identify proteins with unknown sequence processing/PTMs followed by cycles of database filtering and manual sequence adjustment to produce a reduced database with mature protein sequences (i.e. final base amino acid sequences resulting from RNA transcript processing and enzymatic cleavage of terminal amino acids). For the final absolute mass search against the reduced database, ProSight parameters were set as follows: precursor mass tolerance—500 Da; fragment mass tolerance—20 ppm. For High–High searches, only Xtract was used with the same parameters for deconvolution of spectra in both full MS and MS/MS scans.

For validation of unreported PTMs and sequence-processing events, custom scripts were used to combine replicate MS/MS scans for each proteoform with distinct precursor masses prior to assigning fragments. Intensities of assigned masses were *z*-scored, i.e. the intensity divided by the standard deviation after subtracting the mean. The same approach was employed to characterize abundant peaks not identified by the automated searches. Data visualization was done in R with the ggplot2 package^67^. To generate proteoform abundance plots and Supplementary Data 1, monoisotopic or average masses of proteoforms were taken from a list of identified precursor masses and matched against deconvoluted mass features from the full MS-only LC–MS experiments with a mass tolerance window of ±2 Da. Then, all the identified mass features were binned in 3 Da mass windows allowing to filter out the proteoforms present in <4 technically replicate runs (out of 6 total runs). Consequently, protein abundances were calculated as the sum of proteoform fractional abundances (i.e. summed intensity of all charge states normalized on total ion intensity of the LC–MS run).

### Optimization of crosslinking conditions

Crosslink reaction conditions were optimized within the range of 0.5–5 mM for DSSO and 1–50 mM for EDC on PSII–LHCIIsc purified from plants grown in C light. DSSO was considered as a “long-range” crosslinker, with a spacer arm of ~11.3 Å and reactive groups targeting primary amines (lysine and amino termini of proteins). This reagent provides distance constraints between ~7 and ~31 Å considering the flexibility of lysine side-chains (7 Å + 7 Å) and the α-carbon backbone (6 Å). EDC was considered as a “short-range” crosslinker, lacking a spacer arm and with reactive groups targeting carboxylic acids and primary amines (aspartic and glutamic acids to lysine and amino termini of proteins). This reagent provides distance constraints between 0 and 17 Å considering the flexibility of the side-chains (7 and 5 Å for lysine and carboxylic acids, respectively) and the α-carbon backbone (6 Å). The distance cut-off for DSSO crosslinks was previously increased up to 35 Å considering overall protein flexibility^68^, and an average cut-off distance of 33–35 Å was experimentally determined considering the solvent accessible surface distance^49^. In this work, even considering the high degree of intrinsic PSII–LHCIIsc flexibility, we decided to consider a strict 33 Å cut-off distance for the long-range crosslinker DSSO.

PSII–LHCIIsc, at a final protein concentration of 1 mg/mL in a buffer made of 20 mM MES pH 5.7, 0.65 M Sucrose, 10 mM NaCl, 5 mM MgCl_2,_ were crosslinked for 30 and 120 min with DSSO and EDC, respectively. Each reaction was performed at 4 °C in the dark and without stirring to minimize induced conformational changes and unspecific aggregation. The reaction was then quenched with 100 mM Tris–HCl pH 8. Unspecific aggregation was investigated either by denaturing SDS–PAGE^69^, or by non-denaturing lpBN–PAGE^60^ (see Supplementary Fig. 5). The occurrence of one clear band in the region corresponding to the mass of paired PSII–LHCIIsc above the 2 MDa MW marker in the lpBN–PAGE further supports the absence of unspecific aggregation and that EDC, and partially DSSO, are able to maintain the paired conformation during the electrophoretic run. Once the optimal protein:crosslinker ratio was determined, 125 µg of two independently isolated PSII–LHCIIsc from each condition (L, C and H) were pooled and crosslinked either with DSSO or EDC at their optimal final concentration of 1 and 35 mM, respectively. To remove pigments and sucrose, compounds potentially interfering with LC–MS analysis, samples were precipitated by stepwise addition of twice the volume of ice-cold acetone every 30 s while shaking to reach a final protein:acetone ratio of 1:8 (v/v) and incubated overnight at −20 °C. Samples were centrifuged at 15,000 × *g* for 15 min at 4 °C and the acetone completely poured of prior to digestion. Tryptic digestion was conducted by a two-step workflow where the first step was performed at 1:50 ratio (protein:protease) at twice the initial volume (i.e. protein concentration of 0.5 mg/mL in 250 µL) directly on the protein pellet for 4 h at 37 °C constantly shaking to loosen the protein pellet. Pre-digested proteins were denatured by addition of Urea and Thiourea to a final concentration of 4 and 1 M, respectively. After resuspension of the pellet under gentle shaking (~2 h), reduction with 10 mM Dithiothreitol for 1 h at 37 °C, and alkylation with 20 mM Iodoacetamide for 30 min at room temperature in the dark was performed. The second tryptic digestion was performed at 1:25 ratio (protein:protease) for 16 h at 37 °C, after diluting the Urea down to 1 M, and then quenched with 10% Trifluoroacetic acid. The final peptide mixtures were desalted with C_18_ Sep-Pak cartridges (Waters). The crosslinking reaction on thylakoid membranes was performed on three independent samples of 500 µg of total protein isolated from pea plants grown in moderate light (C). Thylakoids were crosslinked for 30 min with 1 mM DSSO at a final protein concentration of 0.5 mg/mL in a buffer composed of 25 mM MES pH 6.0, 10 mM NaCl, 5 mM MgCl_2_. After the crosslinking reaction, the samples were further processed with the same procedure used for PSII–LHCIIsc.

### Fractionation of crosslinked peptides and LC–MS/MS

Fractionation of 250 µg of desalted crosslinked peptides was performed by HPLC-SCX (strong cation exchange) chromatography on an Agilent 1200 HPLC system with a C_18_ trap column (Opti-Lynx TRAP column C_18_/49 µm, 5 mm) connected to an analytical PolyLC column (PolySULFOETHYL A/3 µm, 50 × 1.0 mm). Samples were reconstituted in 10% (v/v) formic acid in water and separated on the analytical column with a 65 min linear gradient from Buffer A (20% (v/v) ACN and 0.05% (v/v) formic acid in water) to 90% Buffer B (20% (v/v) ACN and 0.05% (v/v) formic acid in a 0.5 M NaCl)^70^. The fractions containing crosslinked peptides, from min 14 to 30, were pooled two by two from min 14–21 and 29–30, while the others were kept separate. This resulted in 11 fractions for each sample, which were desalted with C_18_ 96-well elution plates (Oasis HLB), lyophilized and resuspended in 10% Formic acid prior to injection. For measurement, the fractions were injected and separated on a 50 cm × 75 µm C_18_ analytical column, packed in-house (Poroshell 120 EC-C_18_/2.7 µm) connected to Agilent 1290 LC system and an Orbitrap Fusion Tribrid Mass Spectrometer (Thermo Fisher Scientific, Bremen, Germany). Acquisition settings for MS analysis of DSSO crosslinked samples were as previously described^70^ with minor modifications. Briefly, for DSSO crosslinked samples, a survey MS1 scan at high-resolution (60,000) was followed by a Top-N of 10 MS2-CID (collision-induced dissociation) scans on selected high-charged precursors (*z* = 3–8) at resolution of 30,000, producing signature peaks for subsequent MS3 fragmentation of potential crosslinked peptides by higher collisional dissociation (HCD) fragmentation at NCE = 30. For samples crosslinked with EDC, a survey MS1 scan at high-resolution (60,000) was followed by a Top-N of 10 MS2-HCD scans on selected high-charged precursors (*z* = 3–8) at a resolution of 30,000.

Raw data were processed with the XlinkX nodes incorporated in Proteome Discoverer 2.3.0.522 (Thermo Fisher Scientific Bremen, Germany). For peptide/protein identification Mascot was used to search filtered spectra against two databases: (1) the transcriptome-derived database merged with sequences of *P. sativum* available on UniProtKB/TrEMBL repository^61^ and, (2) the TD-MS-derived database containing all proteoforms for which a sequence has been confidently determined (Supplementary Data 1). The XlinkX node for the analysis of the DSSO crosslinks was set as follows: enzyme name—trypsin (full); maximum number of missed cleavages—2; minimum peptide length—5 amino acids; minimum and maximum peptide mass—300 and 7000 Da, respectively. Precursor mass tolerance was set to 10 ppm, FTMS fragment mass to 20 ppm. Modifications allowed were carbamidomethylation of cysteines, as a fixed modification, and oxidation of methionines and protein N-terminal acetylation, as variable modifications. For the analysis of EDC crosslinked samples XlinkX was set to focus on K-DE linkage, using the same peptide and crosslinks search parameters as for DSSO. For both crosslinking reagents, the FDR was controlled at 1% by Percolator.

### Structural modelling and crosslink mapping

Structural predictions with I-TASSER^71^ were performed using the amino acid sequences determined by TD-MS for the most abundant sequence variant (Supplementary Data 1) of every protein for which the N-terminus was not resolved in the available high-resolution structure of pea PSII–LHCIIsc (PDB: 5xnl). This includes all LHCII subunits and PSII proteins D1, D2, CP47 and PsbH (Supplementary Data 4). Of the five models generated by I-TASSER for each protein, only the one with the highest score was further considered, despite the very high score of the other models due to the template-based prediction.

The starting model of the paired supercomplex was derived from the cryo-EM map of the (C_2_S_2_M)×2 at 14 Å resolution (EMD-3825), fitted with the high-resolution structure of the C_2_S_2_M_2_ (PDB: 5xnl) devoid of one M-trimer and its specific linker Lhcb6 and duplicated to fit the other moiety of the (C_2_S_2_M)×2 cryo-EM map. Final placement of the predicted structures was done by alignment and substitution in the resulting (C_2_S_2_M)×2 model with matchmaker in Chimera v1.12^72^. The root mean square deviation for each structural alignment, considering pruned atom pairs of the backbone that account for over 90% of the total, was below 1 Å. For Lhcb2, not distinguishable from Lhcb1 because of limited resolution of the LHCII trimers and thus currently not localizable with certainty in any known high-resolution PSII–LHCIIsc structure^15–17^, we assumed one copy per S-trimer based on our TD-MS and absolute quantification results. This generated nine models in total, one for each putative position of one Lhcb2 in both S-trimers present in the C_2_S_2_M (Supplementary Fig. 9, Supplementary Data 4), further doubled to occupy the other moiety of the structure. All the theoretical models were evaluated by scoring as follows. Detected crosslinks were grouped with an in-house script in R generating all possible combinations between repeated subunits in each structural model considered (i.e. in the (C_2_S_2_M)×2 Lhcb1 is present in 12 copies, Lhcb2 in 4 copies, Lhcb3 and Lhcb6 in 2 copies, Lhcb4, Lhcb5 and all PSII core subunits in 4 copies). Crosslinks were then visualized and processed with ChimeraX^73^. The scoring of each of the nine models was based on XlinkX score for each Lhcb2 unique interlink validated within the distance restraint in the model considered. To avoid overestimation of crosslinks occurring several times within the same model (e.g. for the multiple copies of Lhcb1), the sum of XlinkX scores was further weighted by the number of crosslinks fitted in each model (Supplementary Data 5). Circos-XL plots provided in Supplementary Fig. 6 were produced in R with “circlize” package^74^.

### Reporting summary

Further information on research design is available in the Nature Research Reporting Summary linked to this article.

## Supplementary information

Supplementary Information

Description of Additional Supplementary Files

Supplementary Data 1

Supplementary Data 2

Supplementary Data 3

Supplementary Data 4

Supplementary Data 5

Supplementary Data 6

Reporting Summary

## Data Availability

The mass spectrometry raw data and associated databases used in this study have been deposited to the ProteomeXchange Consortium (http://proteomecentral.proteomexchange.org) via the PRIDE partner repository^75^ with the dataset identifier PXD017382. Detailed reference to all the data shown in the manuscript is reported in the Source data file.

## Source Data

Source Data
