# Supplementary material for: How paired PSII–LHCII supercomplexes mediate the stacking of plant thylakoid membranes unveiled by structural mass-spectrometry
Source: Nat Commun. 2020 Mar 13;11:1361. doi: 10.1038/s41467-020-15184-1 (PMC7069969; doi:10.1038/s41467-020-15184-1)
Supplement: Supplementary file 1 — Supplementary Information [file 41467_2020_15184_MOESM1_ESM.pdf]

How paired PSII-LHCII supercomplexes mediate the stacking of plant thylakoid membranes unveiled by structural mass-spectrometry

Albanese *et al.*, Nature Communications 2020.

SUPPLEMENTARY INFORMATION

Supplementary information includes:

Supplementary Figures **1** to **11**
Supplementary Note **1**
Supplementary References

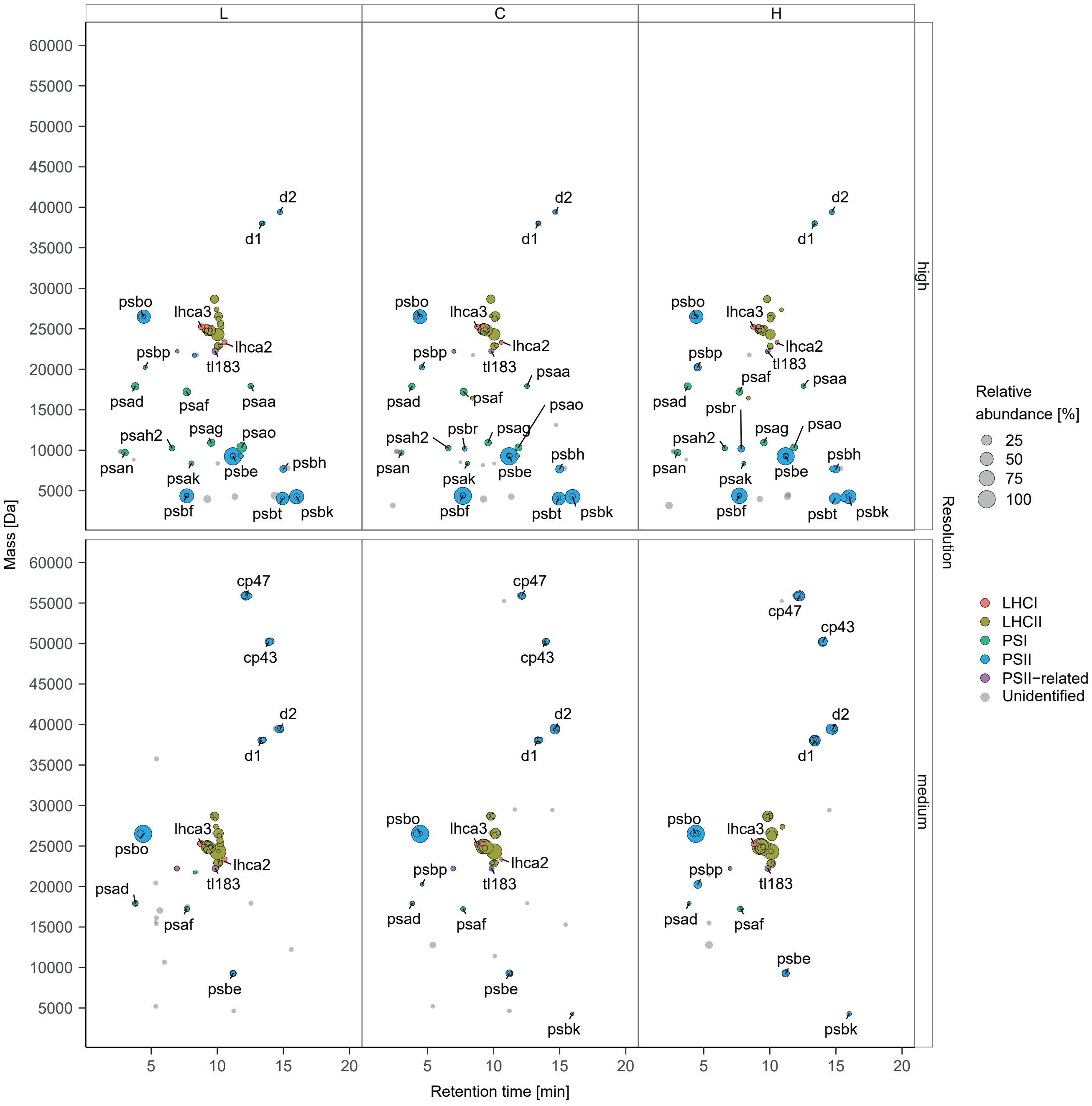

Supplementary Figure 1. Annotated maps of full TD-MS runs of PSII-LHCIIsc samples under different light conditions (L, C, H) measured with either high-resolution (120,000 at 200 *m/z)* or medium-resolution (7,500 at 200 *m/z*) in MS1.

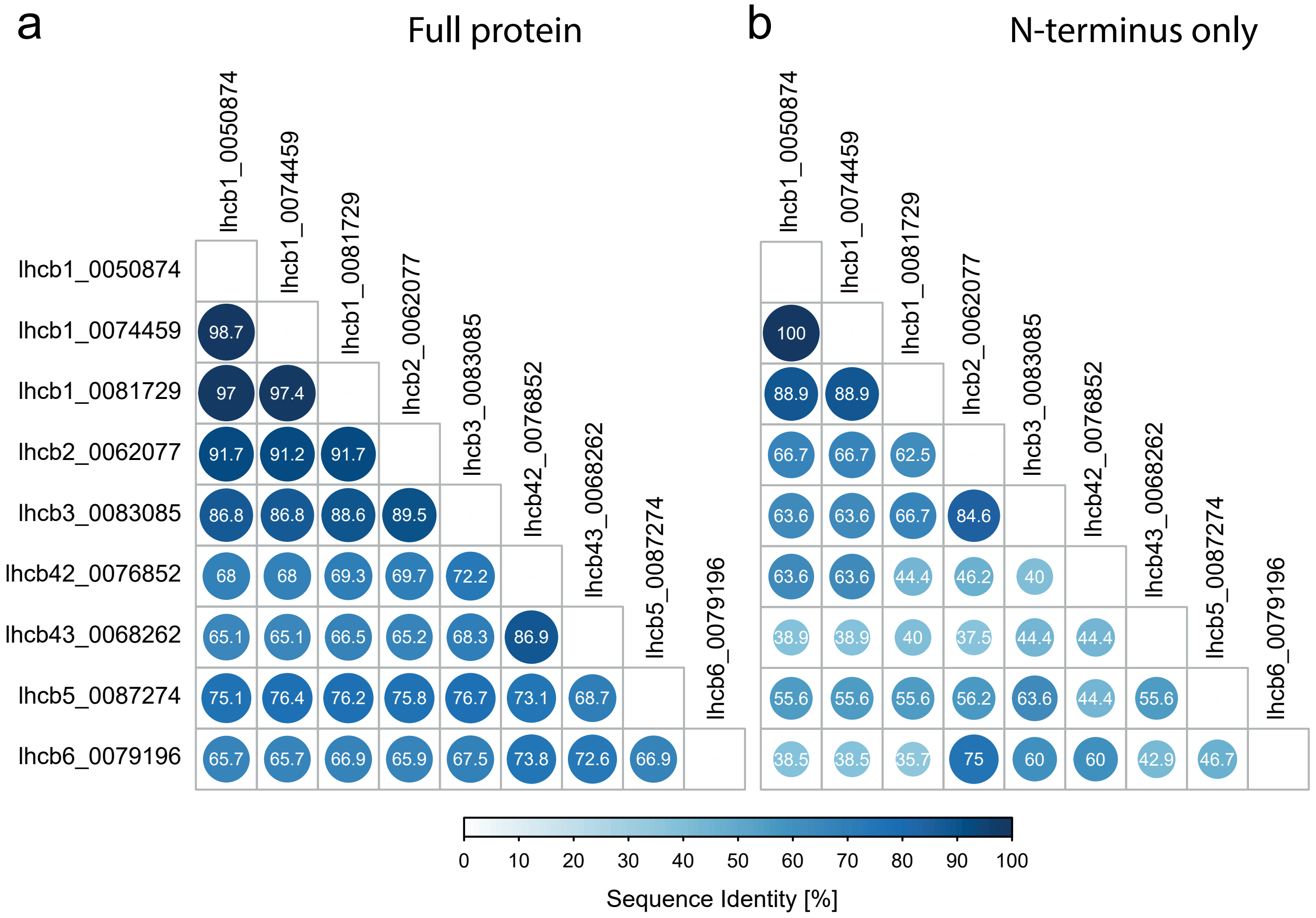

Supplementary Figure 2. Sequence identity between LHCII complete proteoforms detected by TD-MS. Percentages of sequence identity for the full sequences (a) and the first 15 amino acids from the N-terminus (b) were calculated for all LHCII complete (i.e. non-truncated) proteoform sequences in a pairwise manner following multiple sequence alignment using Muscle algorithm.

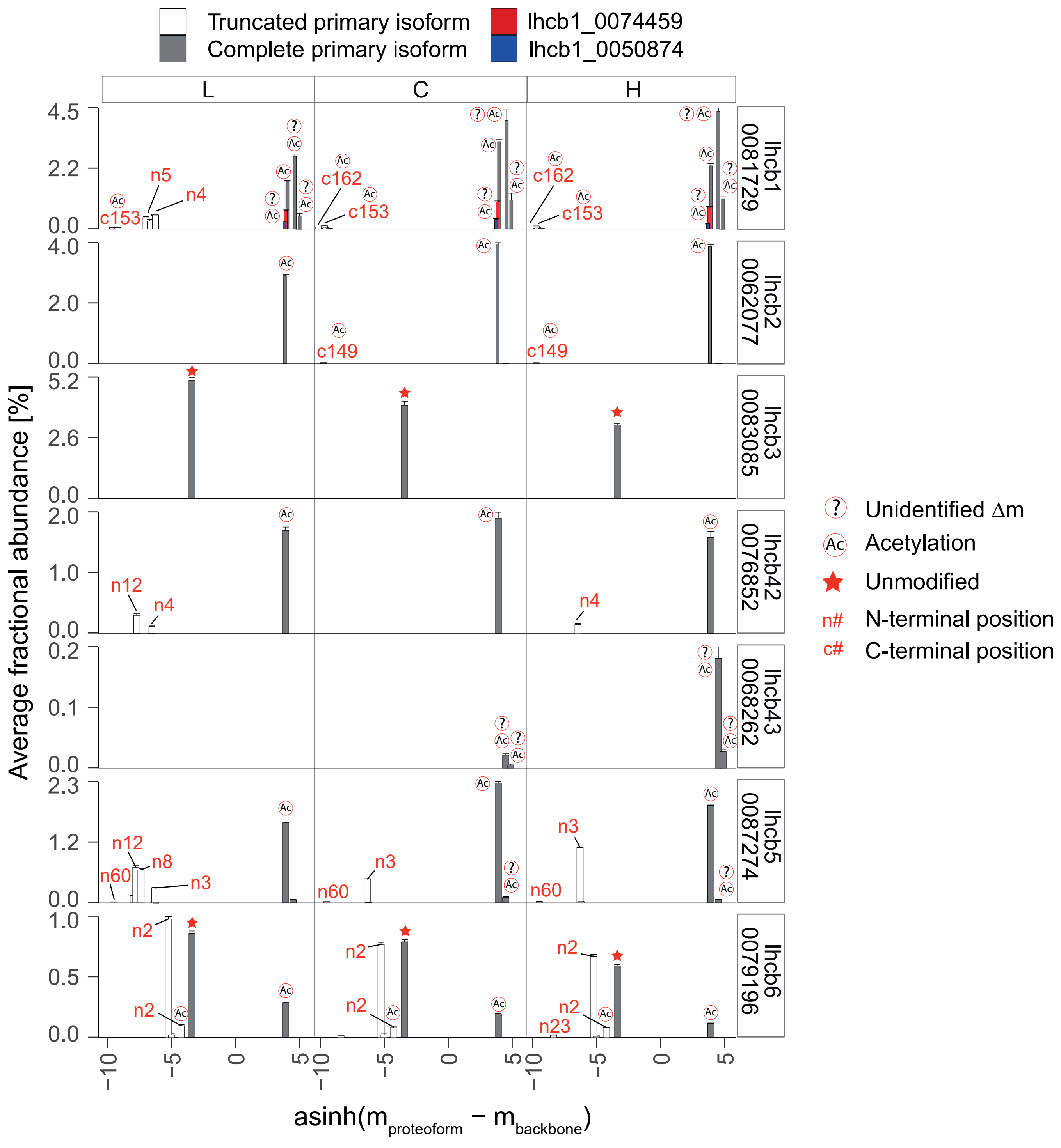

Supplementary Figure 3. Overview of proteoforms identified for LHCII proteins. Each proteoform is represented by the mass difference between experimental mass of the proteoform and the backbone mass of the primary isoform, asinh($m_{proteoform}$ − $m_{backbone}$). For proteoforms with N- or C-terminal truncations, the position of the truncated terminus is indicated as the amino acid residue index in the most abundant sequence for each protein. Acetylation on the N-terminal domain (Ac) of a proteoform was assigned when supported by at least 4 N-terminal fragment ions (see first b-ion in Supplementary Data 1b). Proteoforms containing a mass deviation not fully assigned to PTMs/substitutions/truncations and attributable to a position outside the N-terminus are labelled with encircled question mark.

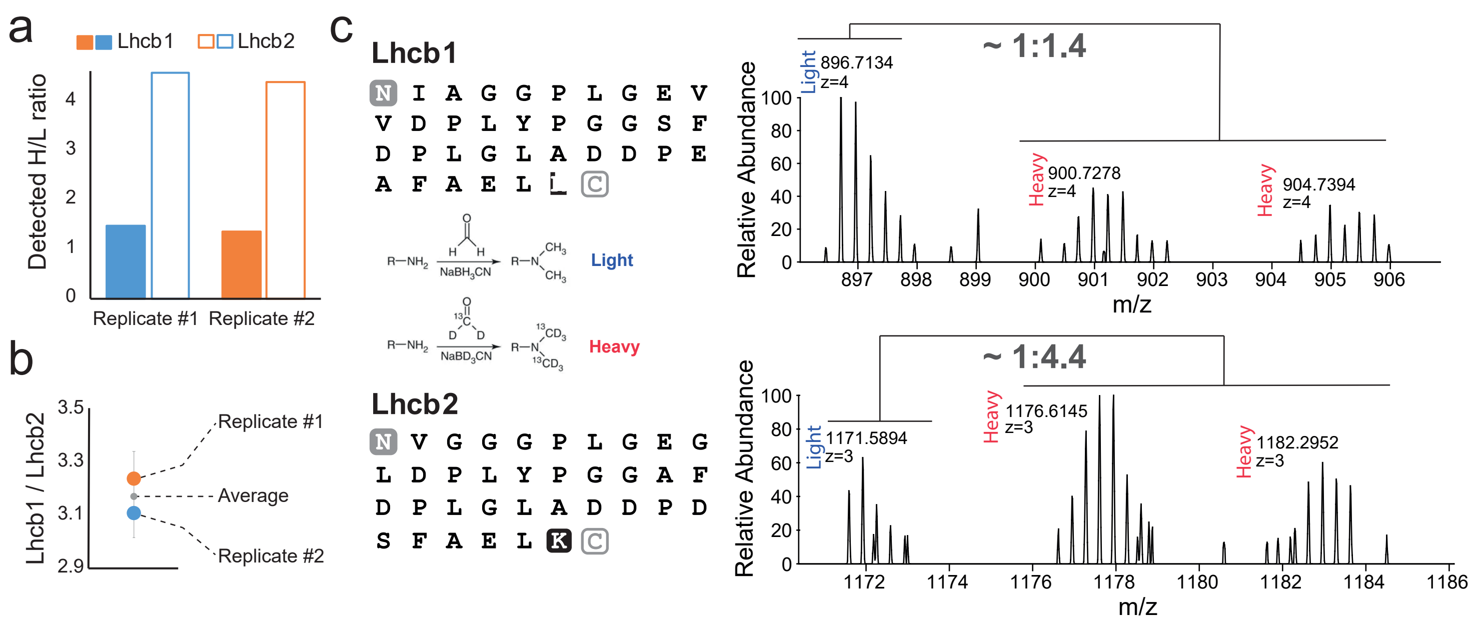

Supplementary Figure 4. Lhcb1/Lhcb2 quantification with labelled synthetic peptides. (a) Relative intensity ratios of 100 nM synthetic peptides (Heavy, H) and endogenous peptides (Light, L) respectively for Lhcb1 (plain bar) and Lhcb2 (empty bar) of the PSII-LHCIIsc. (b) Resulting Lhcb1/Lhcb2 ratios determined for two independent biological replicates. (c) The sequences of the two tryptic peptides quantified with representative MS spectra of each. All quantifications of Lhcb1 and Lhcb2 were made from 19 and 12 MS/MS spectra, respectively, of +3 and +4 charged species.

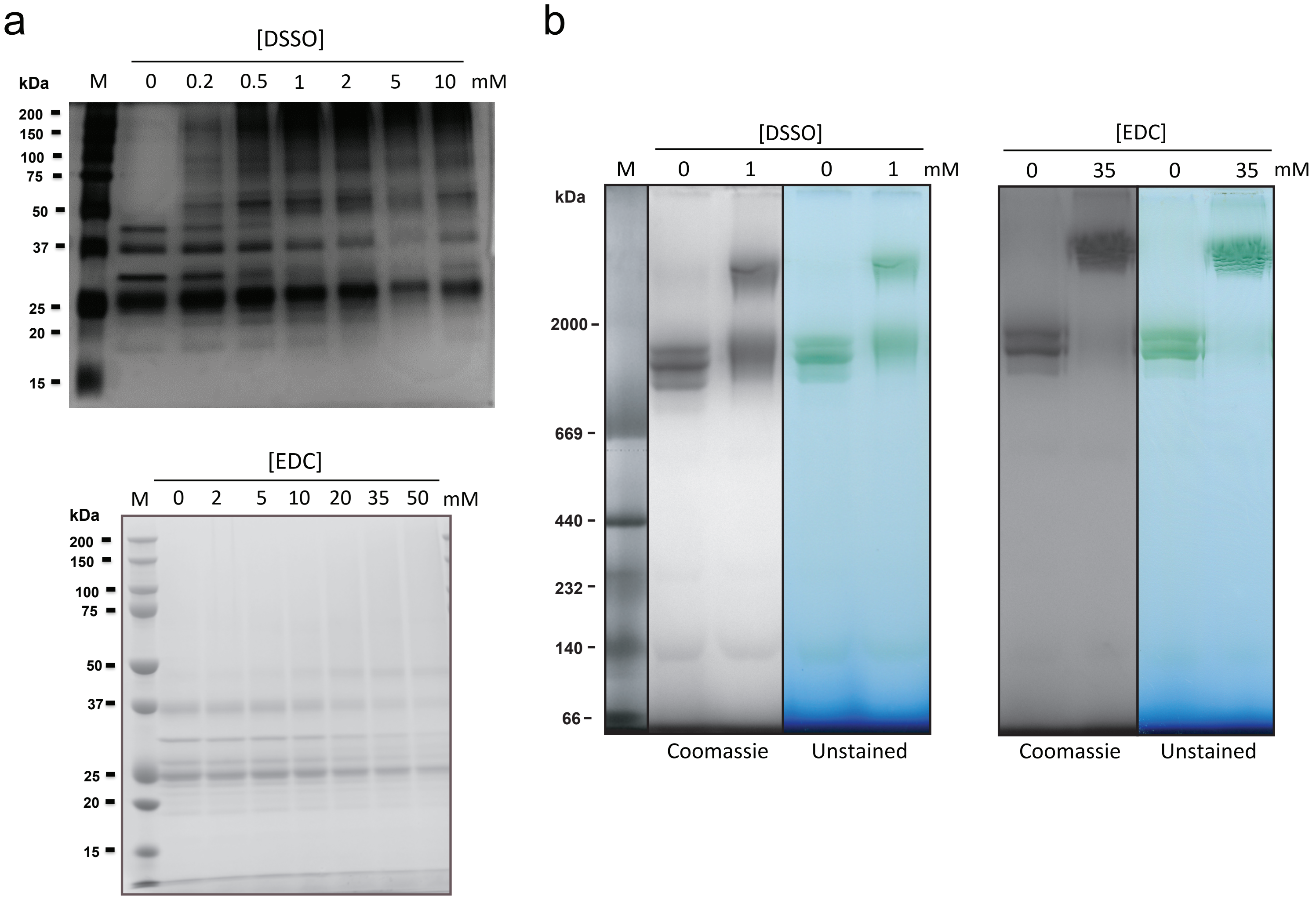

Supplementary Figure 5. Optimization of crosslinking reaction conditions with DSSO and EDC. (a) SDS-PAGE showing the effect of increasing concentrations of DSSO (silver-stained) and EDC (Coomassie-stained) on isolated paired PSII-LHCIIsc and corresponding (b) BN-PAGE separation performed using the optimal concentration of DSSO (1 mM) and EDC (35 mM). Molecular weight marker (M) labeled on the left. 50 µg (SDS-PAGE) and 100 µg (BN-PAGE) of protein were loaded on each lane.

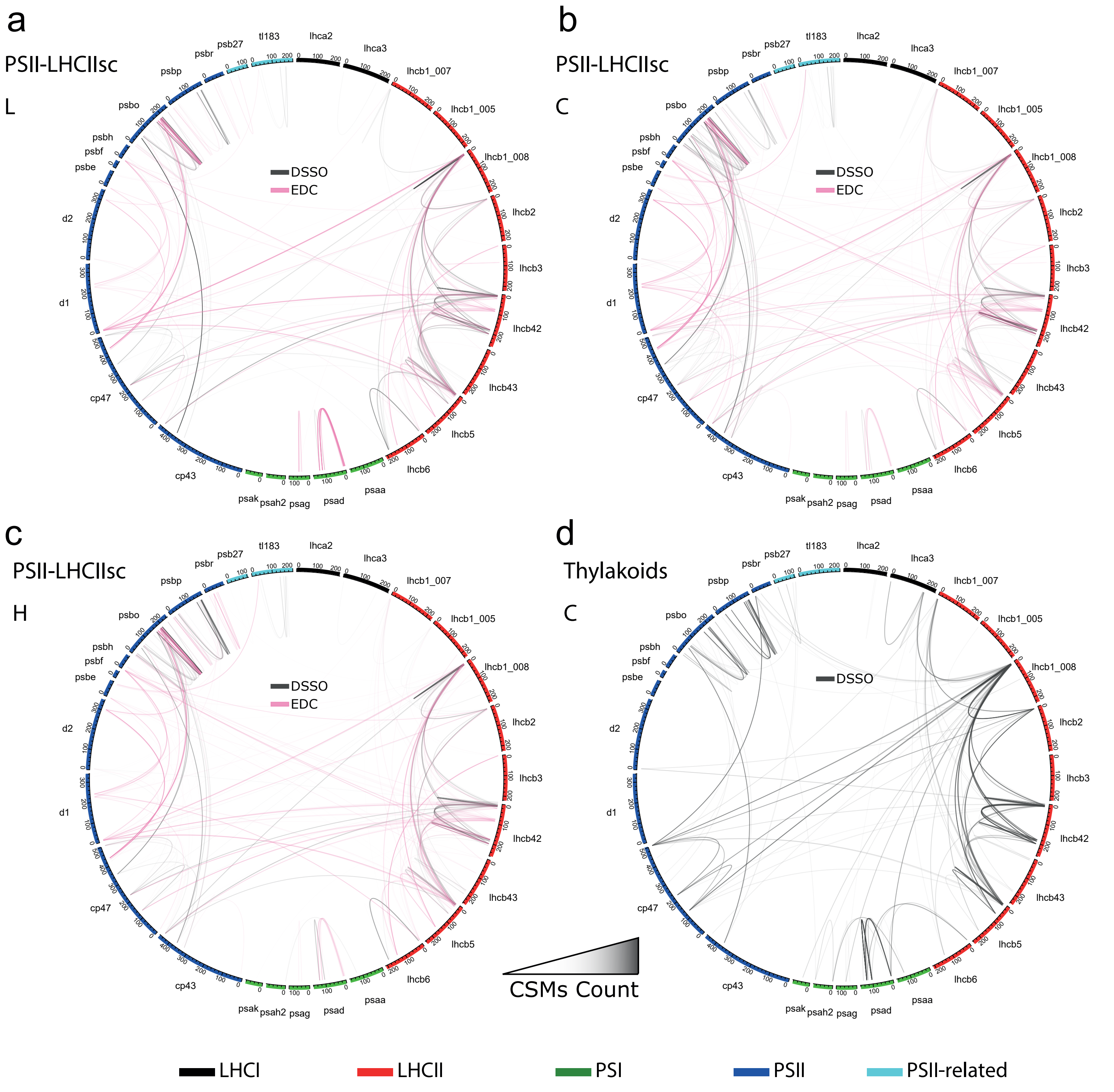

Supplementary Figure 6. Circos-XL plots showing the distribution of all DSSO (grey) and EDC (magenta) crosslinks detected either in PSII-LHCIIsc isolated from plants grown in L (a), C (b) and H (c) light intensities or in the thylakoid membranes isolated from plants grown in C light (d). Each main protein group (i.e. PSII, LHCII, PSI, LHCI and PSII-related proteins) is represented as a colored segment, with the amino acid residue count indicated on the outside of the plot. Line opacity reflects the number of crosslink spectra matches (CSMs) supporting each crosslink.

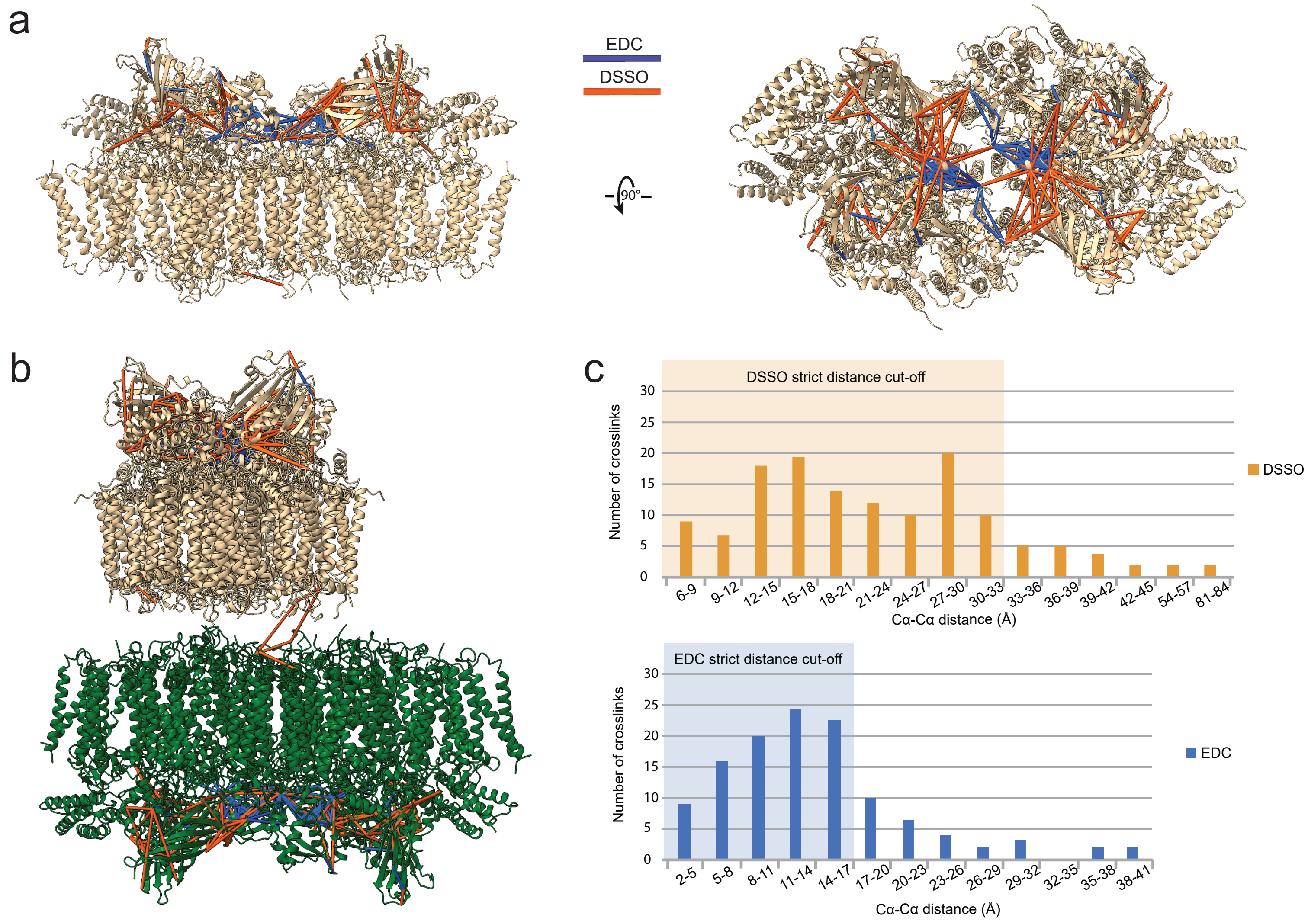

Supplementary Figure 7. Validation of DSSO and EDC XL-MS PSII-LHCIIsc datasets on the PSII core (PDB: 5XNL, [https://www.rcsb.org/structure/5XNL]). (a) Mapping of DSSO (orange) and EDC (blue) crosslinks detected in at least two out of three samples (L, C and H) falling within the strict distance cut-off for each crosslinker (*i.e.* 17 Å for EDC and 33 Å for DSSO). The occurrence of ambiguous crosslinks for DSSO that can be mapped twice, once within the membrane plane and once across the stromal gap, are highlighted in (b). (c) C*α*-C*α* distance distribution of all crosslinks involving PSII core proteins of PDB: 5XNL, [https://www.rcsb.org/structure/5XNL], and their validation according to the strict distance cut-off, or a distance cut-off increased by 1/3.

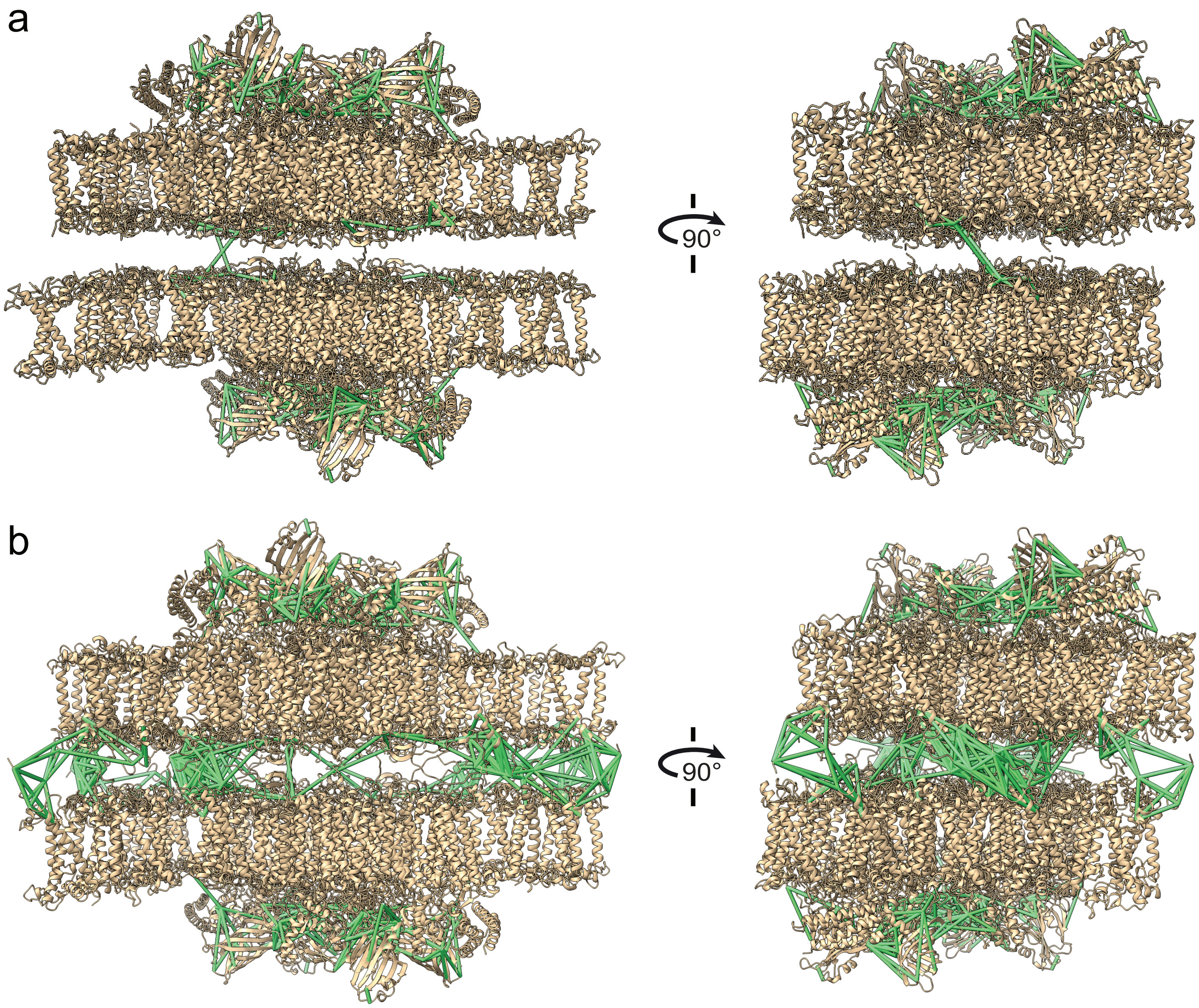

Supplementary Figure 8. Comparison of the two $(C_2S_2M)_{x2}$ structural models generated using either the high-resolution structure available for pea PSII-LHCIIsc (PDB: 5XNL, [https://www.rcsb.org/structure/5XNL]) (a) or the structural predictions using sequences detected by TD-MS for all subunits with incomplete sequence in the corresponding high-resolution structure (PDB: 5XNL, [https://ww-w.rcsb.org/structure/5XNL]) (b). The dataset used is from DSSO crosslinked PSII-LHCIIsc acclimated to moderate light (C sample). The crosslinks visualized (light green lines) are within DSSO distance cut-off (33 Å).

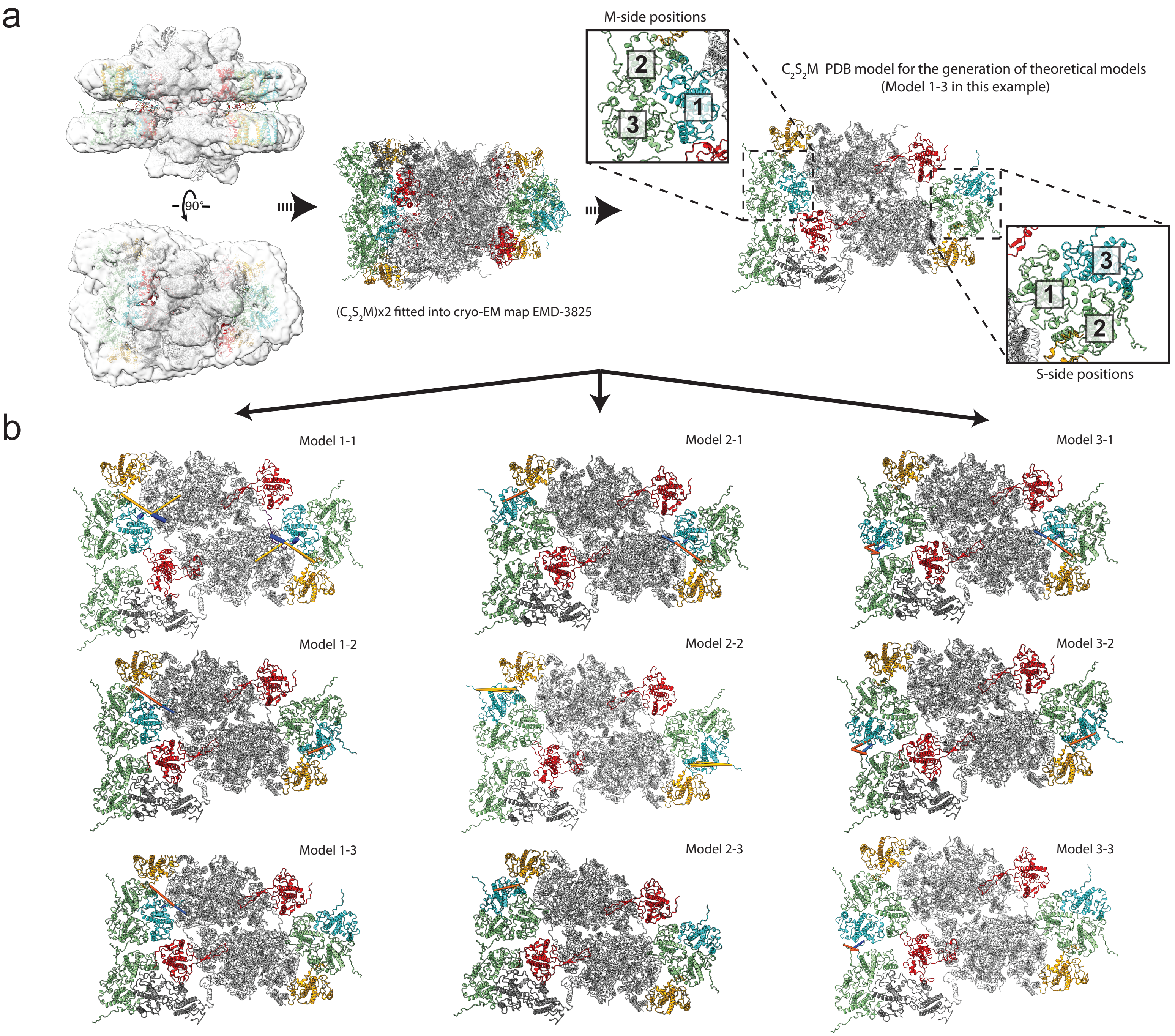

Supplementary Figure 9. Integrative modelling of PSII-LHCIIsc for the positioning of Lhcb2 within the S-trimer. (a) Overview of the workflow for modelling of $(C_2S_2M)x2$ using the PDB: 5XNL, [https://www.rcsb.org/structure/5XNL], with predicted subunits substituted and fitted in the cryo-EM map EMD-3825, [https://www.ebi.ac.uk/pdbe/entry/emdb/EMD-3825], (see Methods for details). Any potential position of Lhcb2 into the S-trimer was numbered as in the example shown and finally generated nine theoretical models of C_2S_2M (b), which were fitted in both moieties of the cryo-EM map. For clarity only the lower C_2S_2M of a $(C_2S_2M)x2$ is shown viewed from the stromal surface.

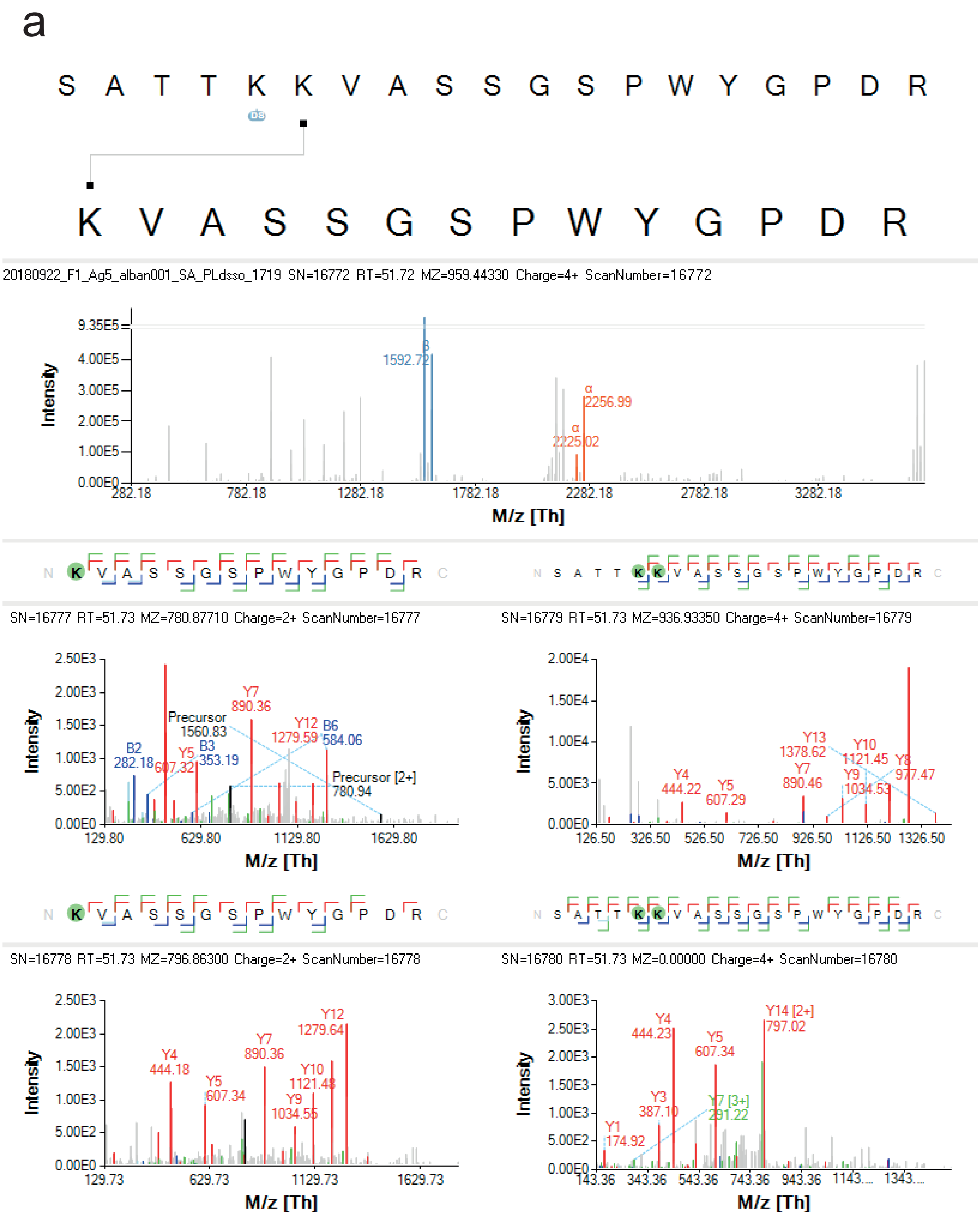

Supplementary Figure 10. MS/MS spectra of the DSSO self-links detected in isolated PSII-LHCIIsc in all three light conditions for Lhcb1 (a-f) and Lhcb4.2 (g-i). Neighboring lysine residues are considered self-links when distance is below 7 Å. When MS2 spectra were not used for identification, the MS3 fragment spectra are shown.

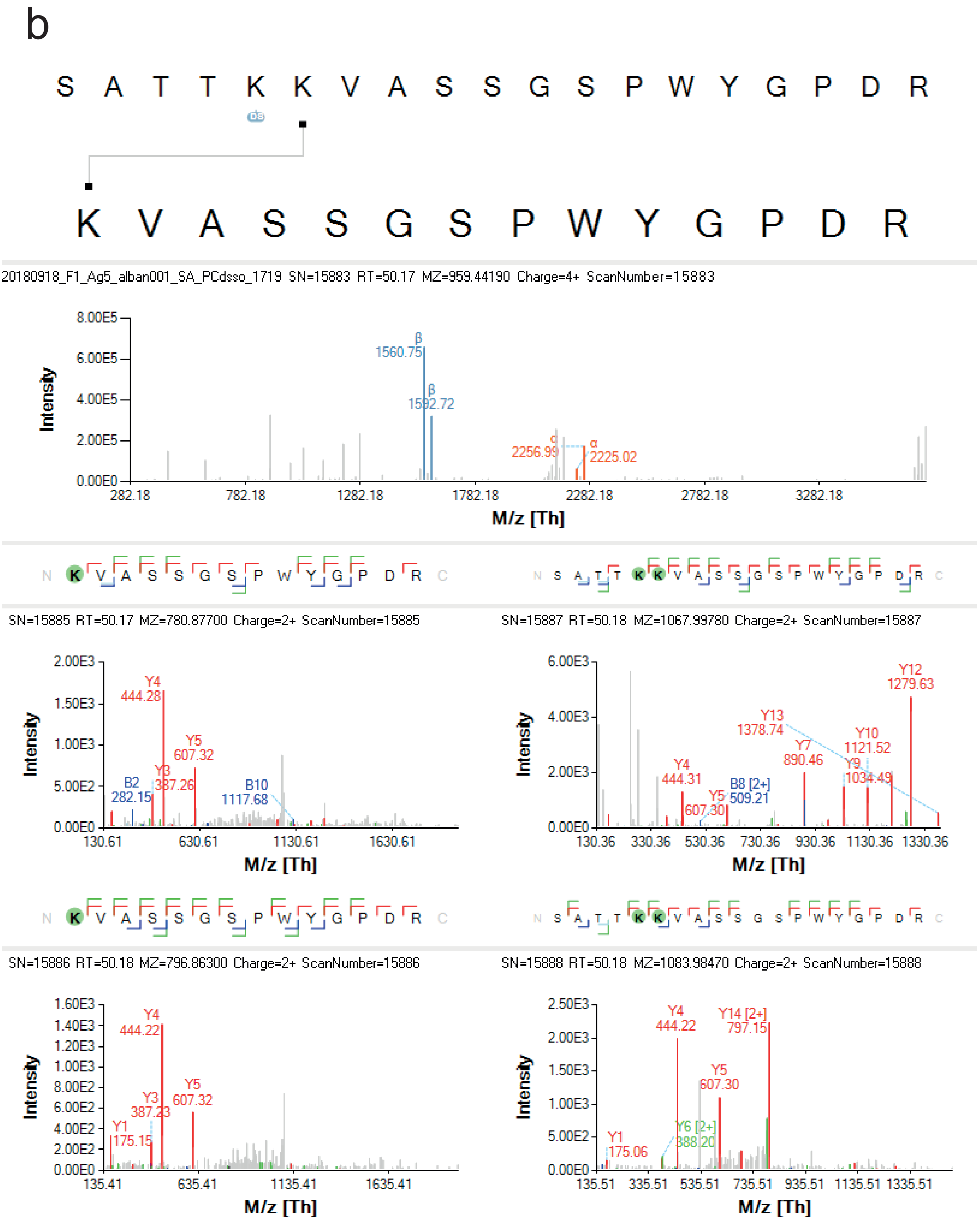

Supplementary Figure 10. MS/MS spectra of the DSSO self-links detected in isolated PSII-LHCIIsc in all three light conditions for Lhcb1 (a-f) and Lhcb4.2 (g-i). Neighboring lysine residues are considered self-links when distance is below 7 Å. When MS2 spectra were not used for identification, the MS3 fragment spectra are shown.

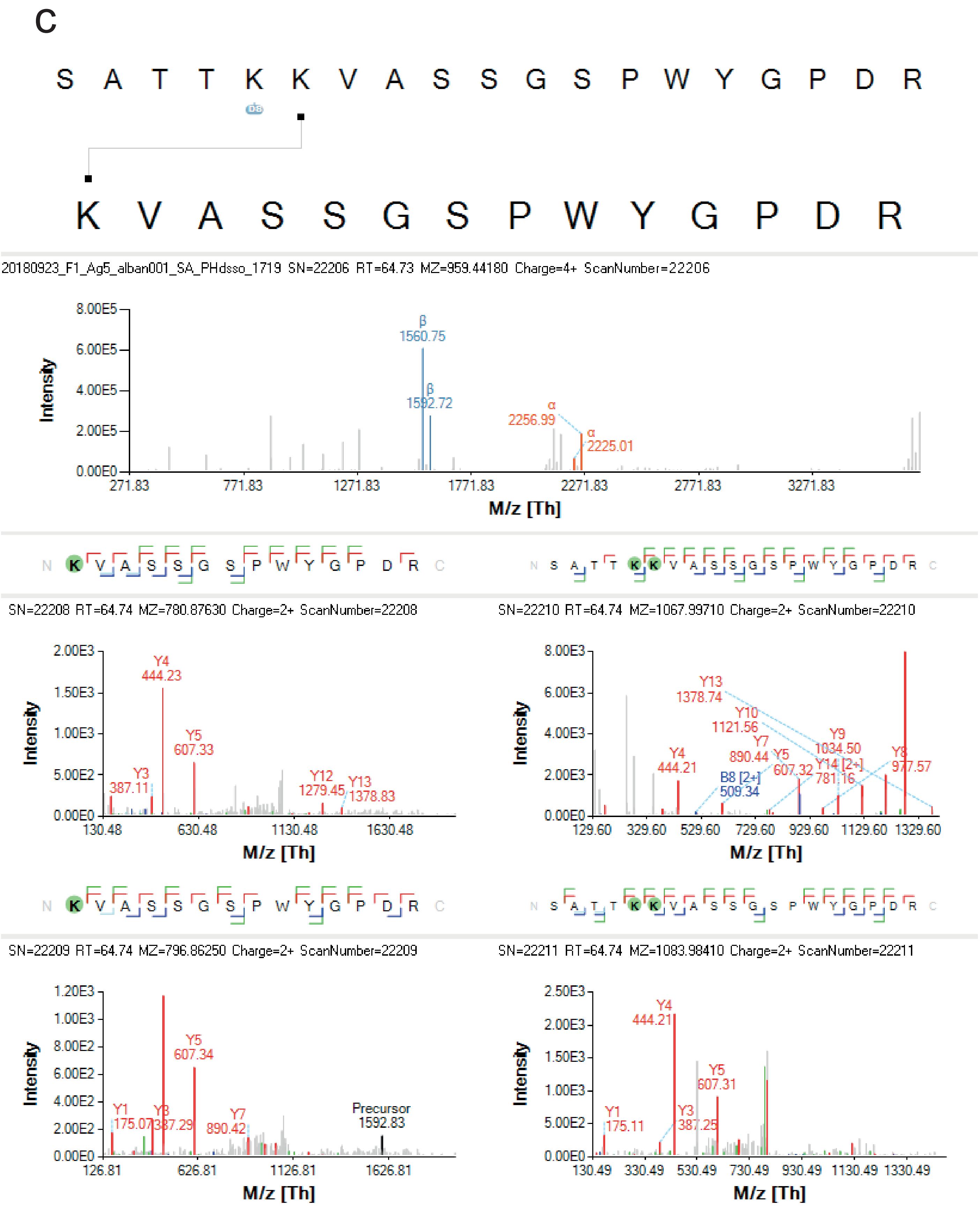

Supplementary Figure 10. MS/MS spectra of the DSSO self-links detected in isolated PSII-LHCIIsc in all three light conditions for Lhcb1 (a-f) and Lhcb4.2 (g-i). Neighboring lysine residues are considered self-links when distance is below 7 Å. When MS2 spectra were not used for identification, the MS3 fragment spectra are shown.

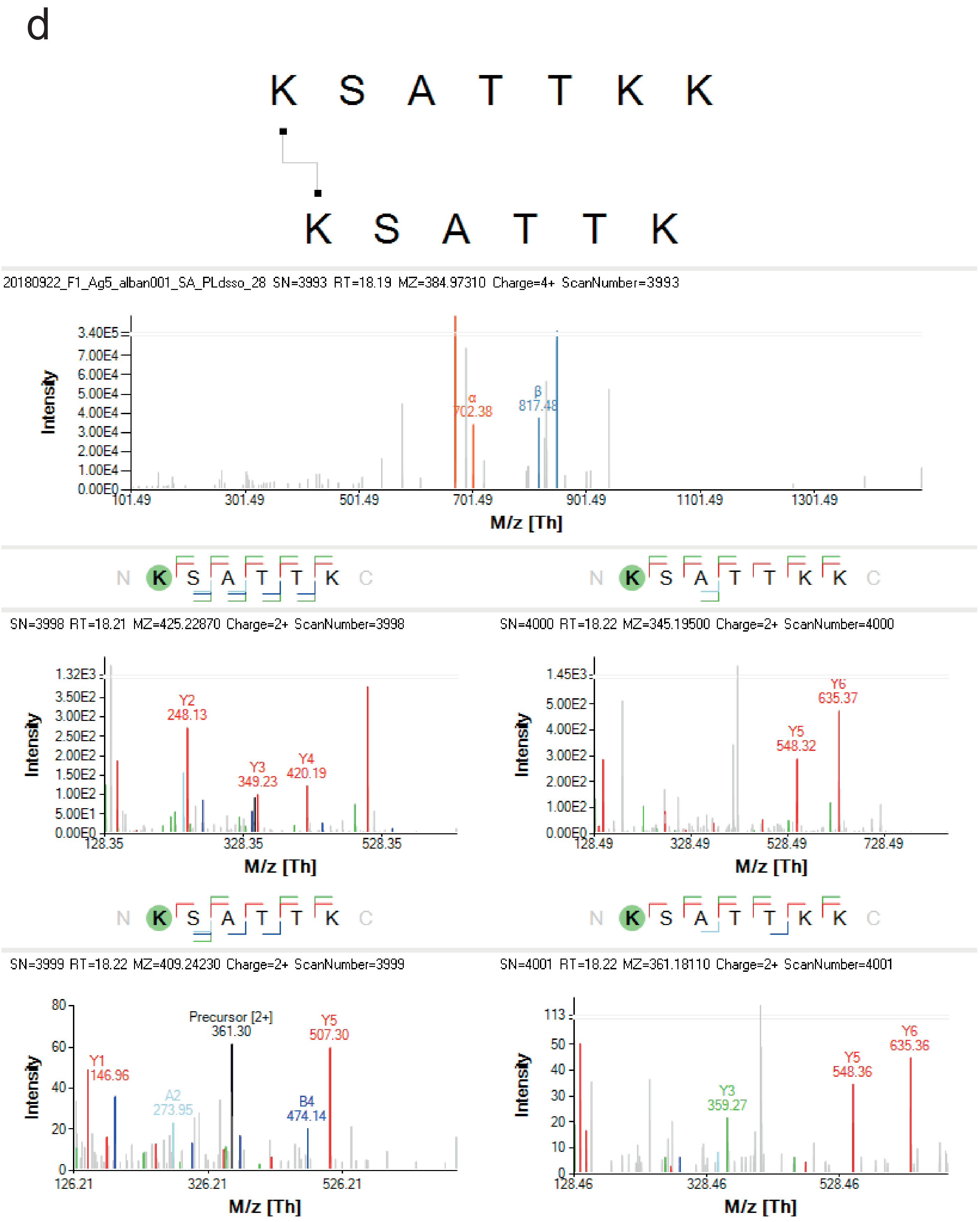

Supplementary Figure 10. MS/MS spectra of the DSSO self-links detected in isolated PSII-LHCIIsc in all three light conditions for Lhcb1 (a-f) and Lhcb4.2 (g-i). Neighboring lysine residues are considered self-links when distance is below 7 Å. When MS2 spectra were not used for identification, the MS3 fragment spectra are shown.

e

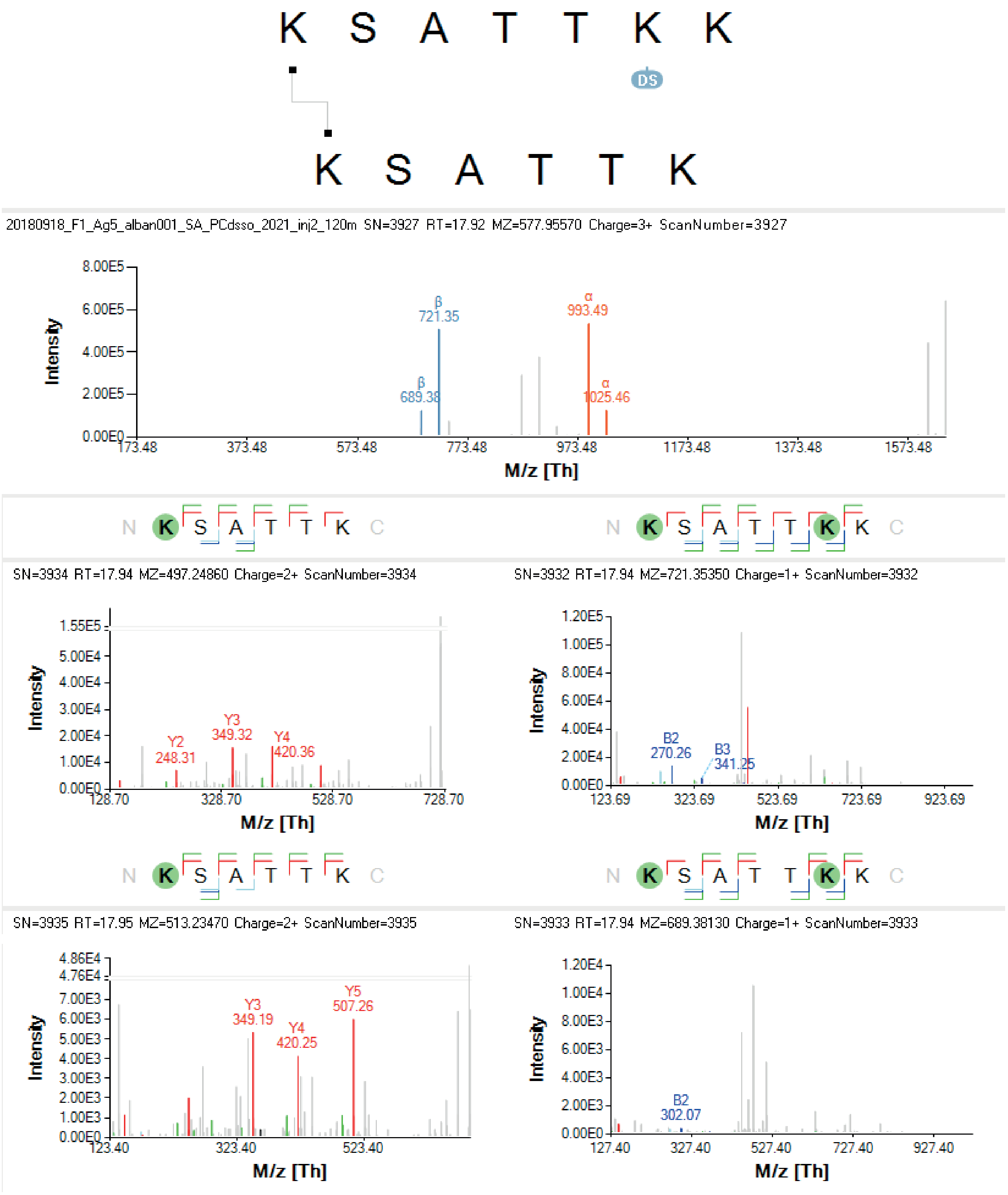

Supplementary Figure 10. MS/MS spectra of the DSSO self-links detected in isolated PSII-LHCIIsc in all three light conditions for Lhcb1 (a-f) and Lhcb4.2 (g-i). Neighboring lysine residues are considered self-links when distance is below 7 Å. When MS2 spectra were not used for identification, the MS3 fragment spectra are shown.

f

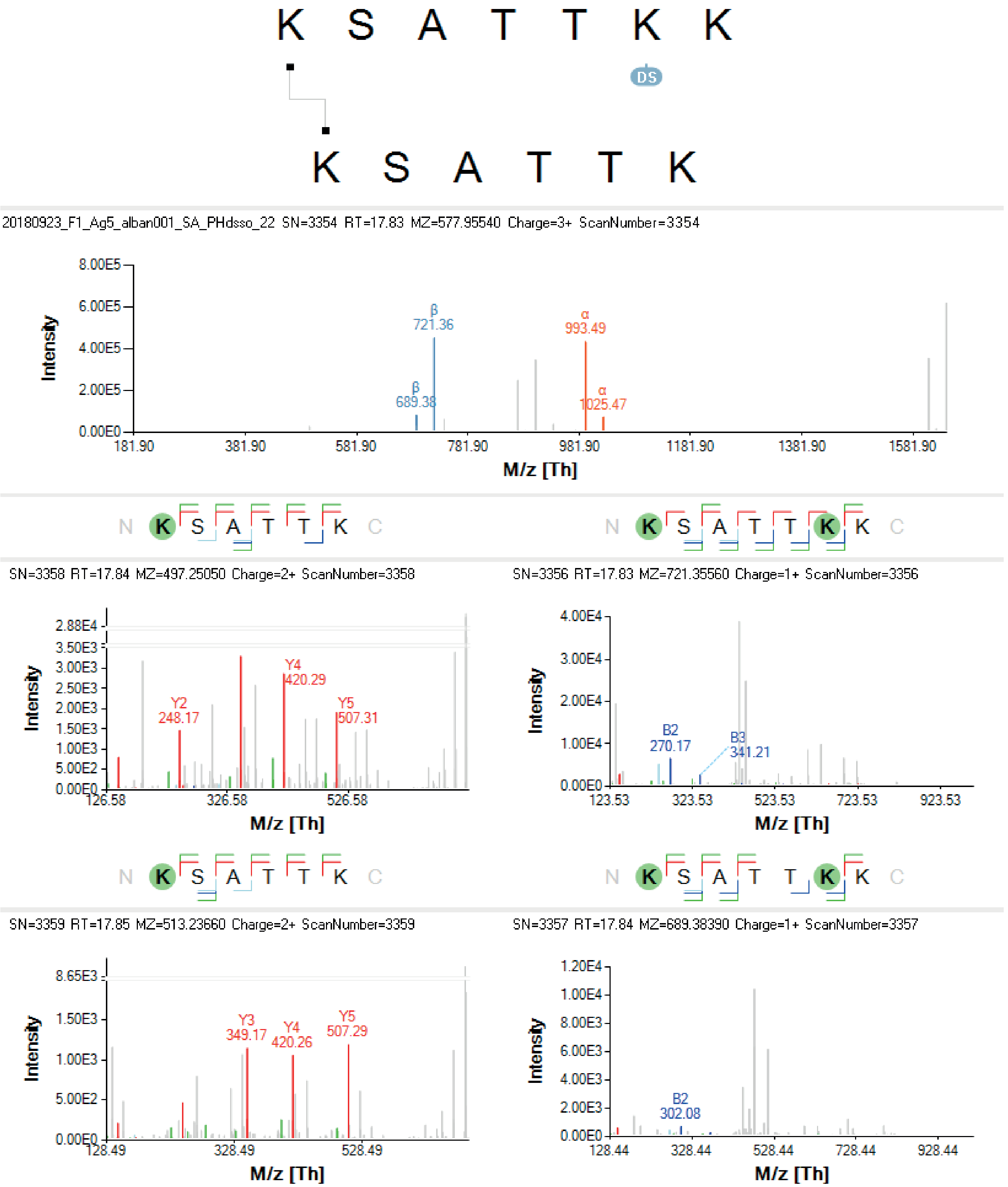

Supplementary Figure 10. MS/MS spectra of the DSSO self-links detected in isolated PSII-LHCIIsc in all three light conditions for Lhcb1 (a-f) and Lhcb4.2 (g-i). Neighboring lysine residues are considered self-links when distance is below 7 Å. When MS2 spectra were not used for identification, the MS3 fragment spectra are shown.

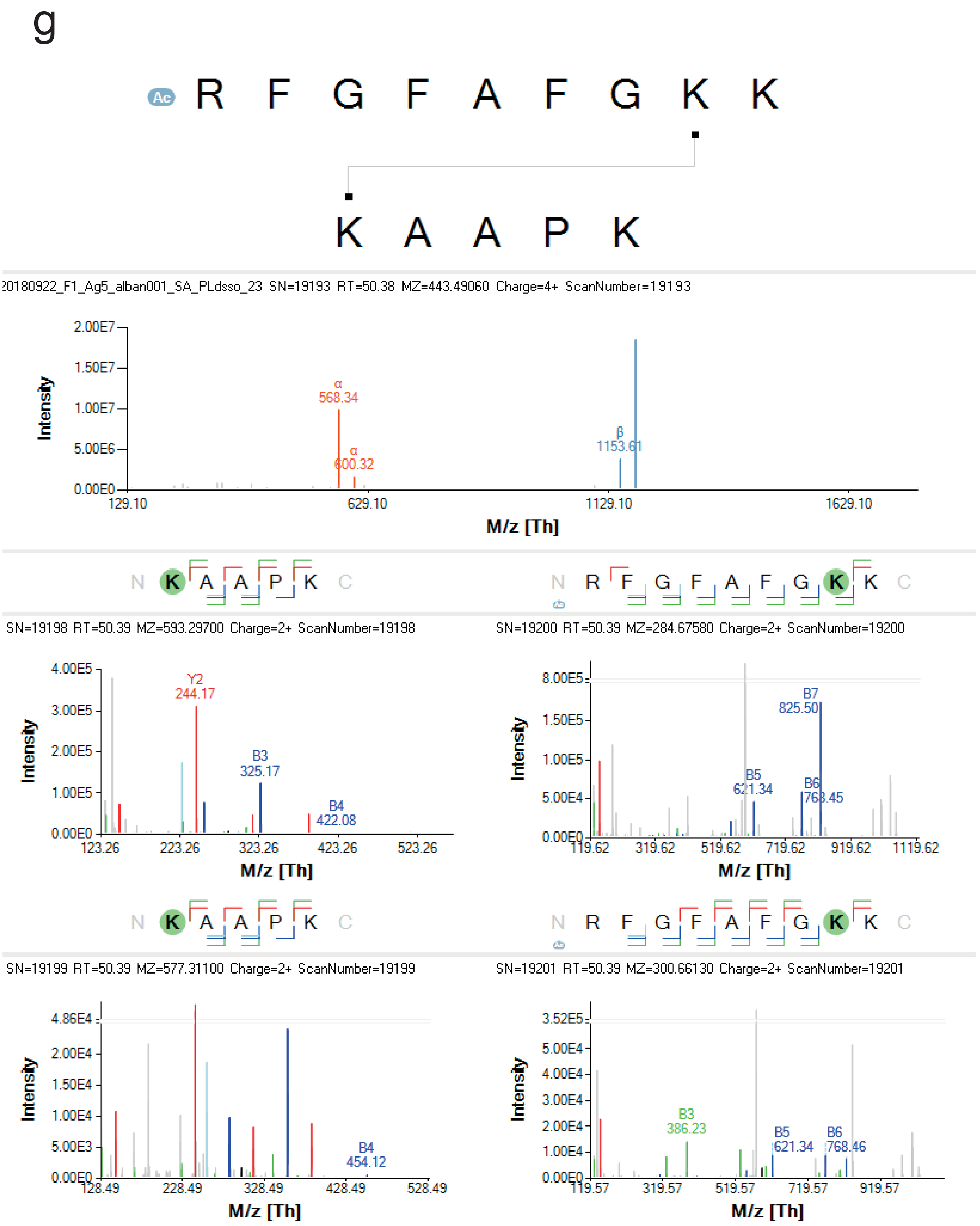

Supplementary Figure 10. MS/MS spectra of the DSSO self-links detected in isolated PSII-LHCIIsc in all three light conditions for Lhcb1 (a-f) and Lhcb4.2 (g-i). Neighboring lysine residues are considered self-links when distance is below 7 Å. When MS2 spectra were not used for identification, the MS3 fragment spectra are shown.

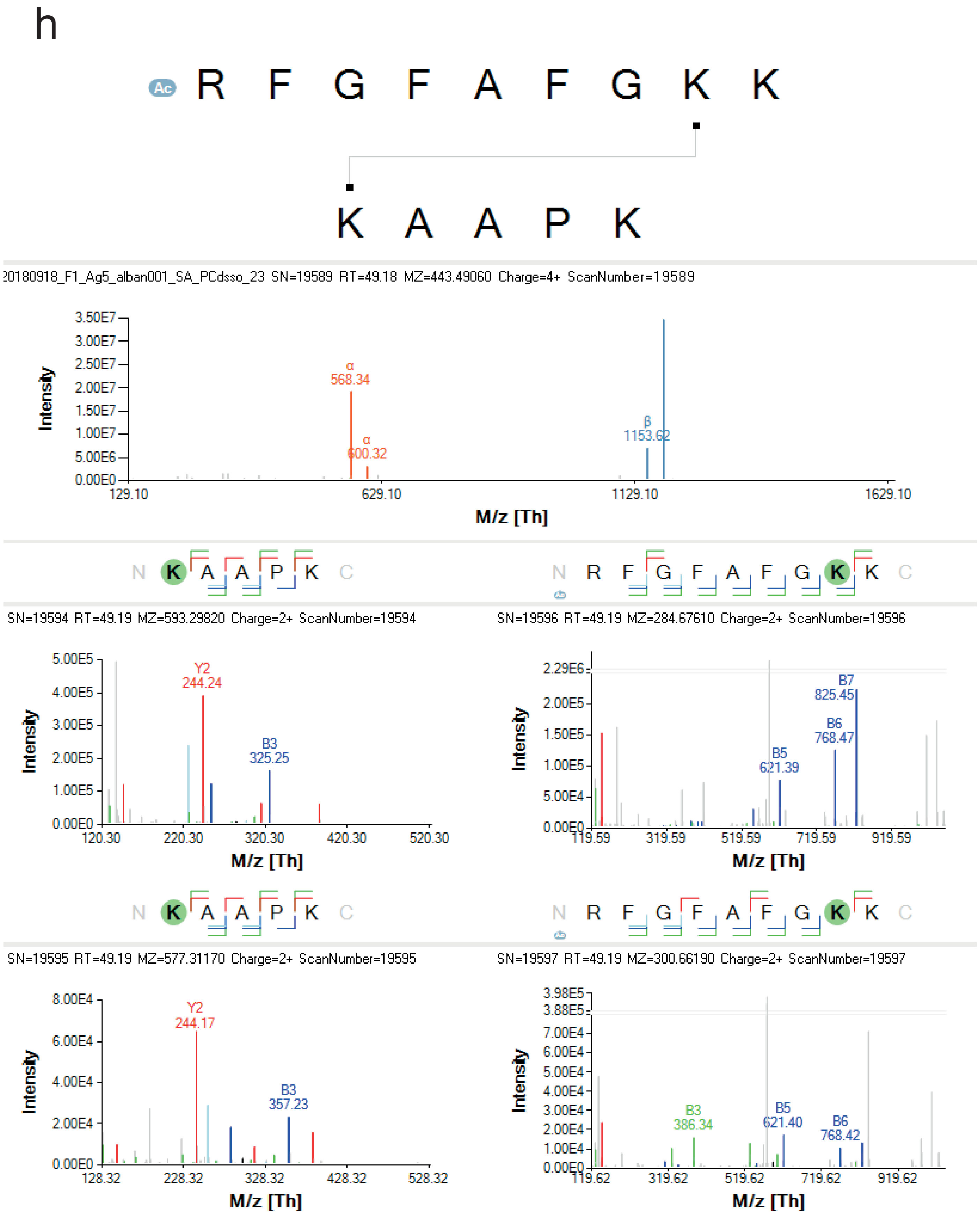

Supplementary Figure 10. MS/MS spectra of the DSSO self-links detected in isolated PSII-LHCIIsc in all three light conditions for Lhcb1 (a-f) and Lhcb4.2 (g-i). Neighboring lysine residues are considered self-links when distance is below 7 Å. When MS2 spectra were not used for identification, the MS3 fragment spectra are shown.

i

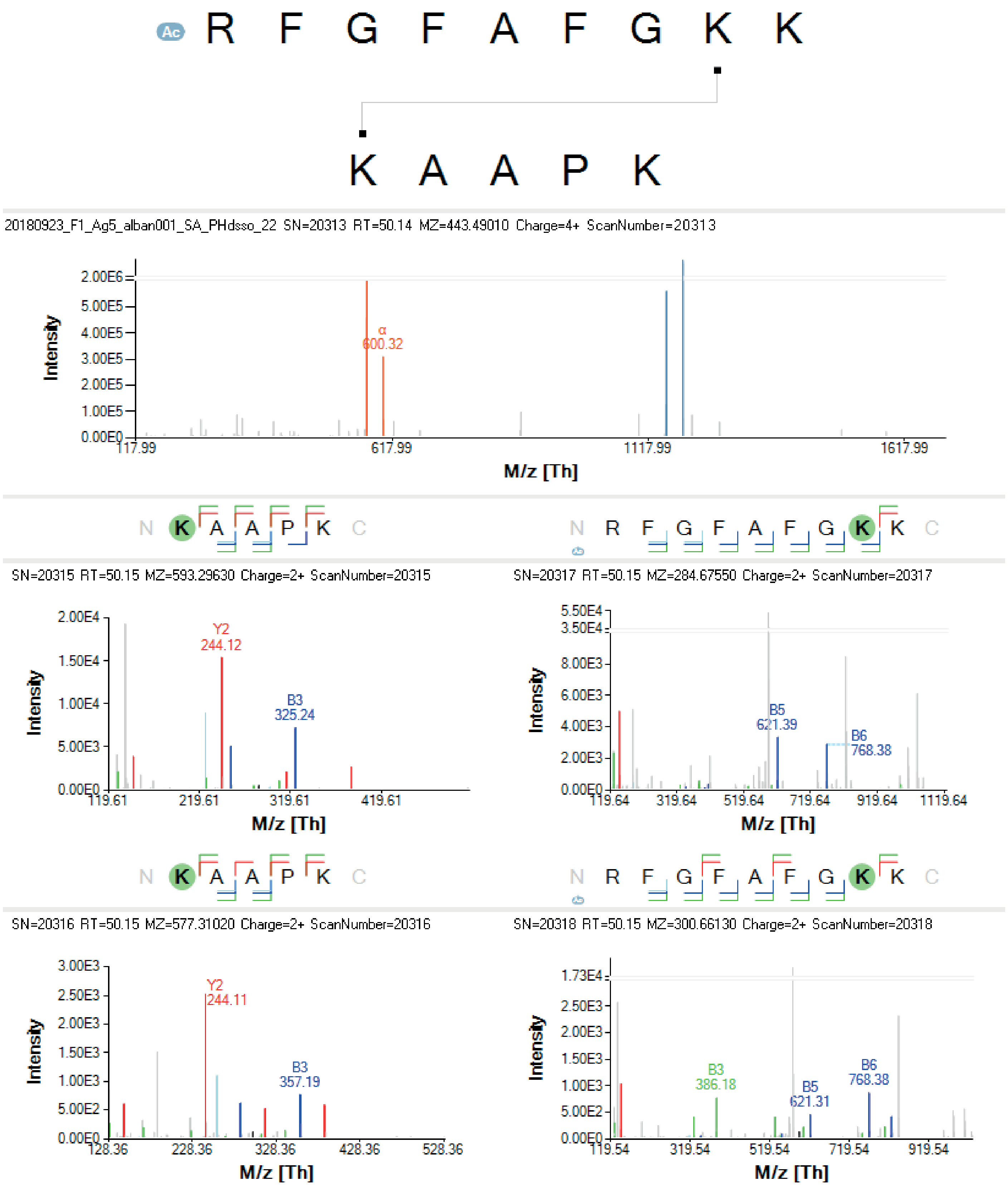

Supplementary Figure 10. MS/MS spectra of the DSSO self-links detected in isolated PSII-LHCIIsc in all three light conditions for Lhcb1 (a-f) and Lhcb4.2 (g-i). Neighboring lysine residues are considered self-links when distance is below 7 Å. When MS2 spectra were not used for identification, the MS3 fragment spectra are shown.

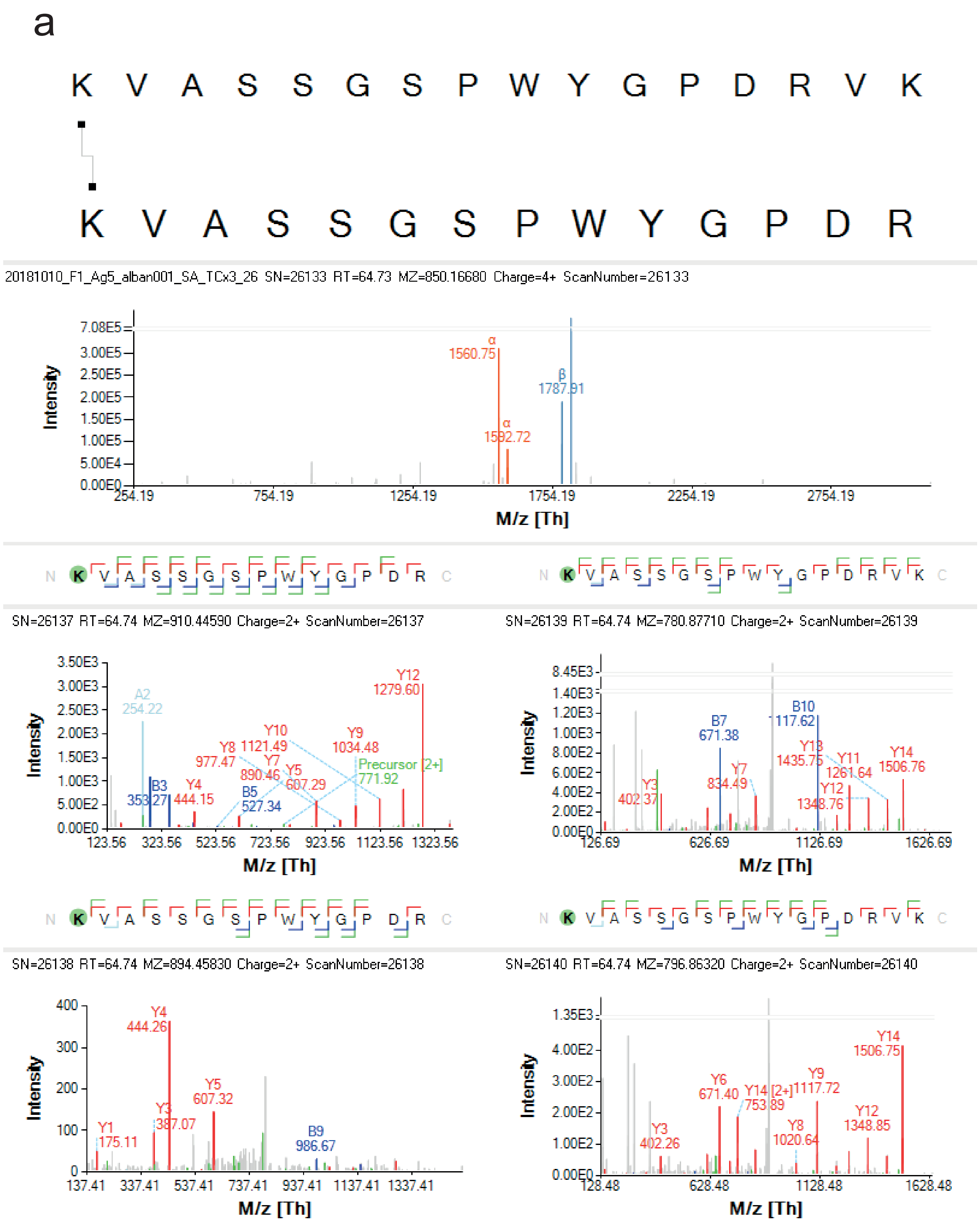

Supplementary Figure 11. MS/MS spectra of the DSSO self-links detected in thylakoid membranes for Lhcb1 (a-c) and Lhcb4.2 (d-f). Neighboring lysine residues are considered self-links when distance is below 7 Å. When MS2 spectra were not used for identification, the MS3 fragment spectra are shown.

b

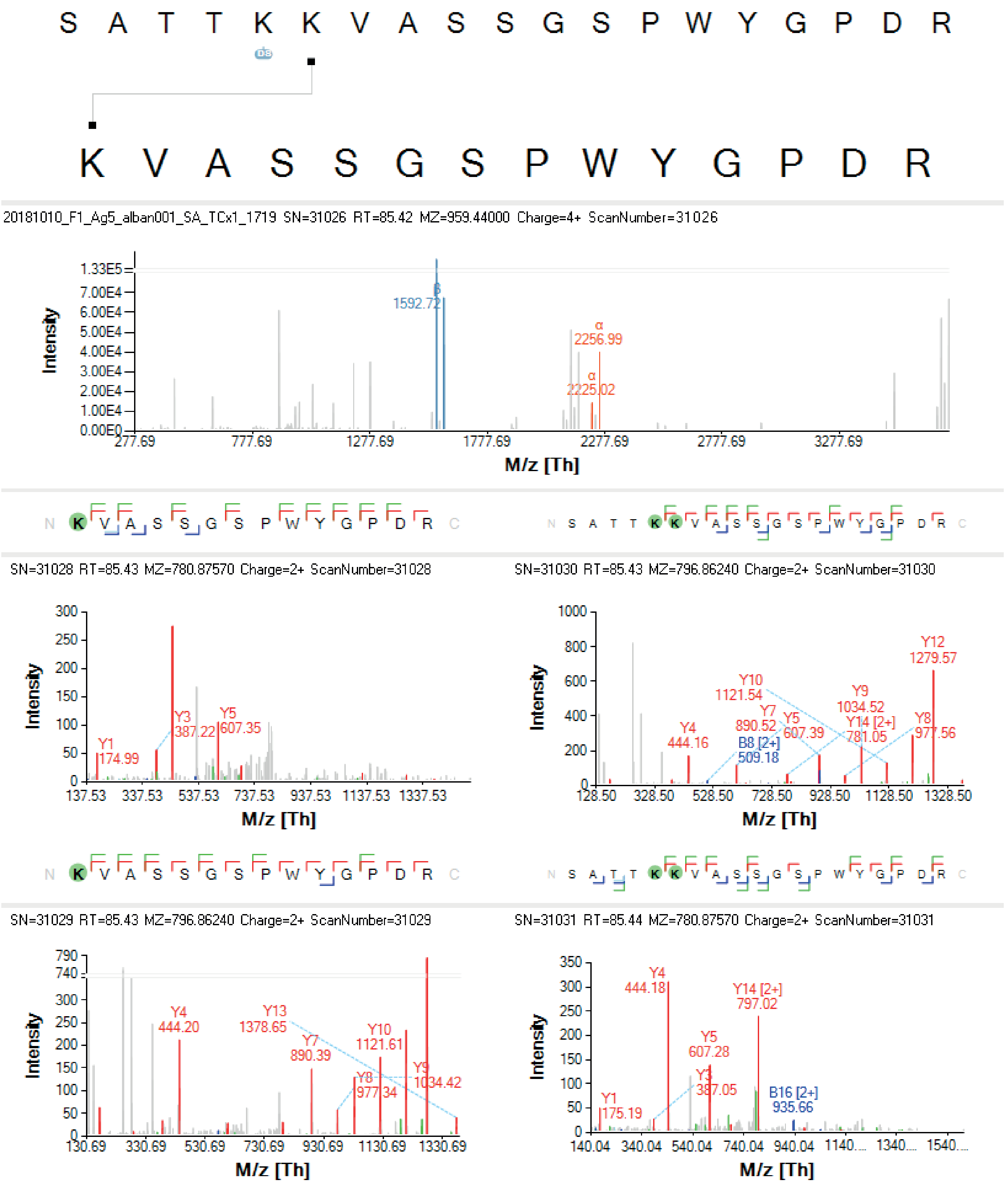

Supplementary Figure 11. MS/MS spectra of the DSSO self-links detected in thylakoid membranes for Lhcb1 (a-c) and Lhcb4.2 (d-f). Neighboring lysine residues are considered self-links when distance is below 7 Å. When MS2 spectra were not used for identification, the MS3 fragment spectra are shown.

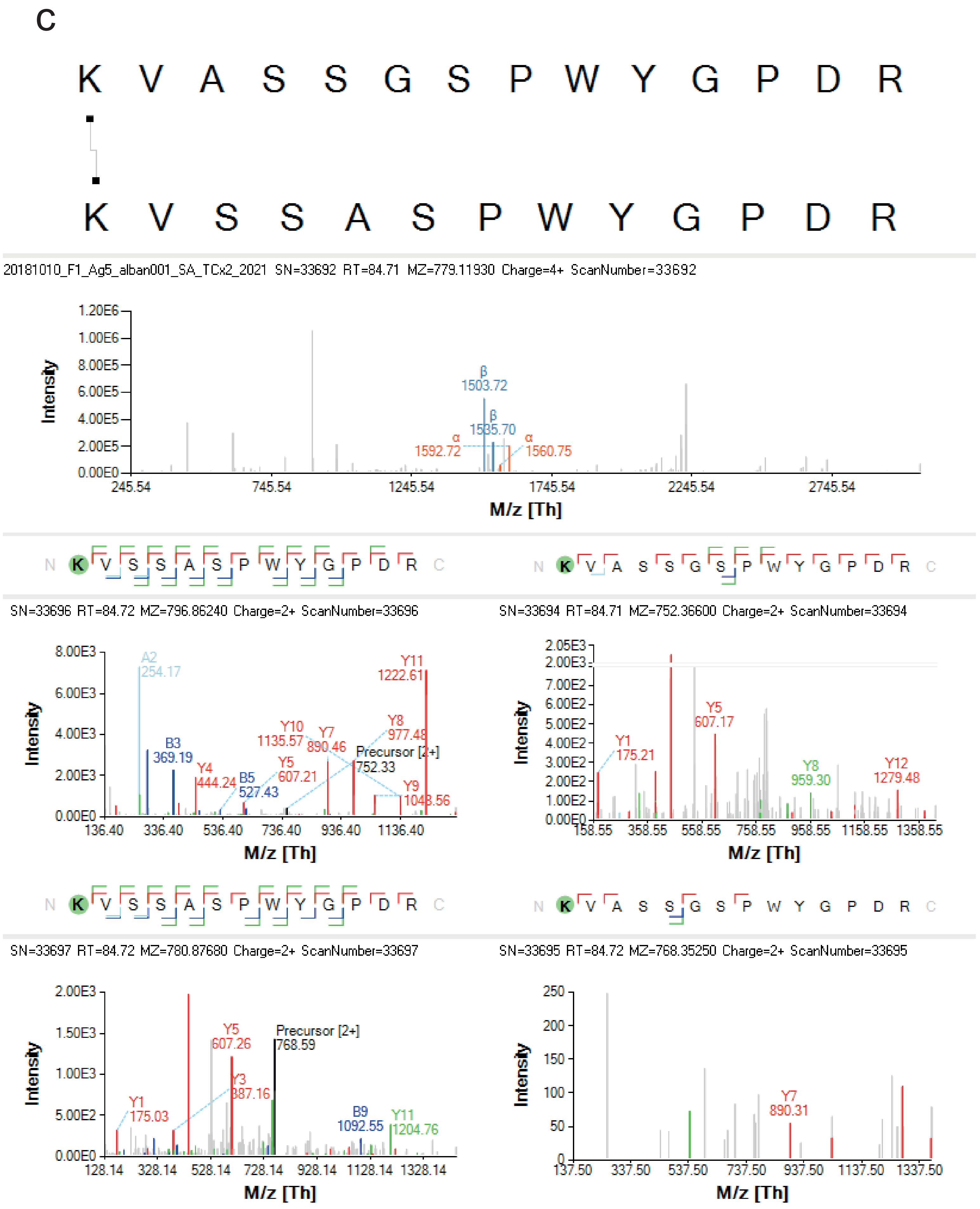

Supplementary Figure 11. MS/MS spectra of the DSSO self-links detected in thylakoid membranes for Lhcb1 (a-c) and Lhcb4.2 (d-f). Neighboring lysine residues are considered self-links when distance is below 7 Å. When MS2 spectra were not used for identification, the MS3 fragment spectra are shown.

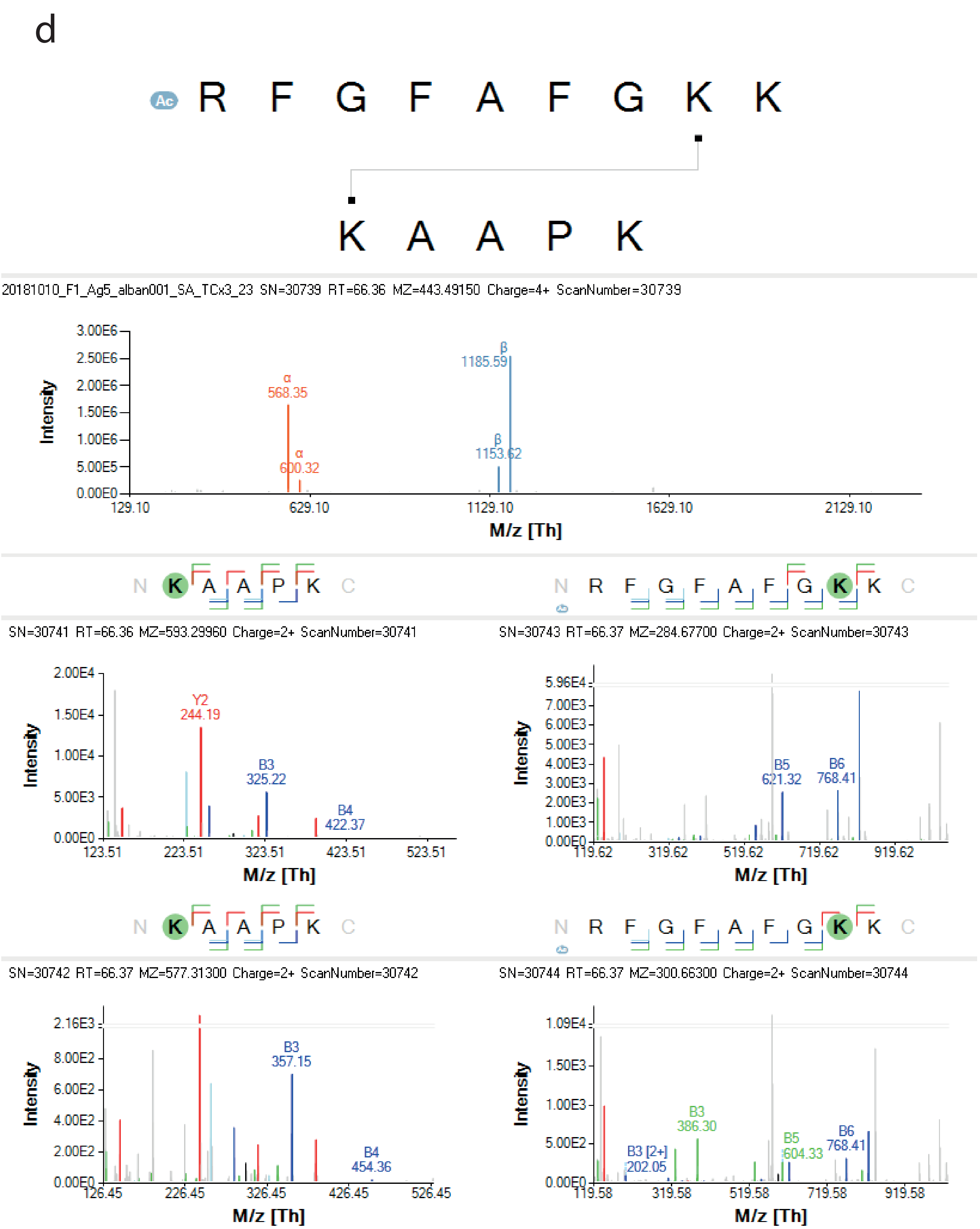

Supplementary Figure 11. MS/MS spectra of the DSSO self-links detected in thylakoid membranes for Lhcb1 (a-c) and Lhcb4.2 (d-f). Neighboring lysine residues are considered self-links when distance is below 7 Å. When MS2 spectra were not used for identification, the MS3 fragment spectra are shown.

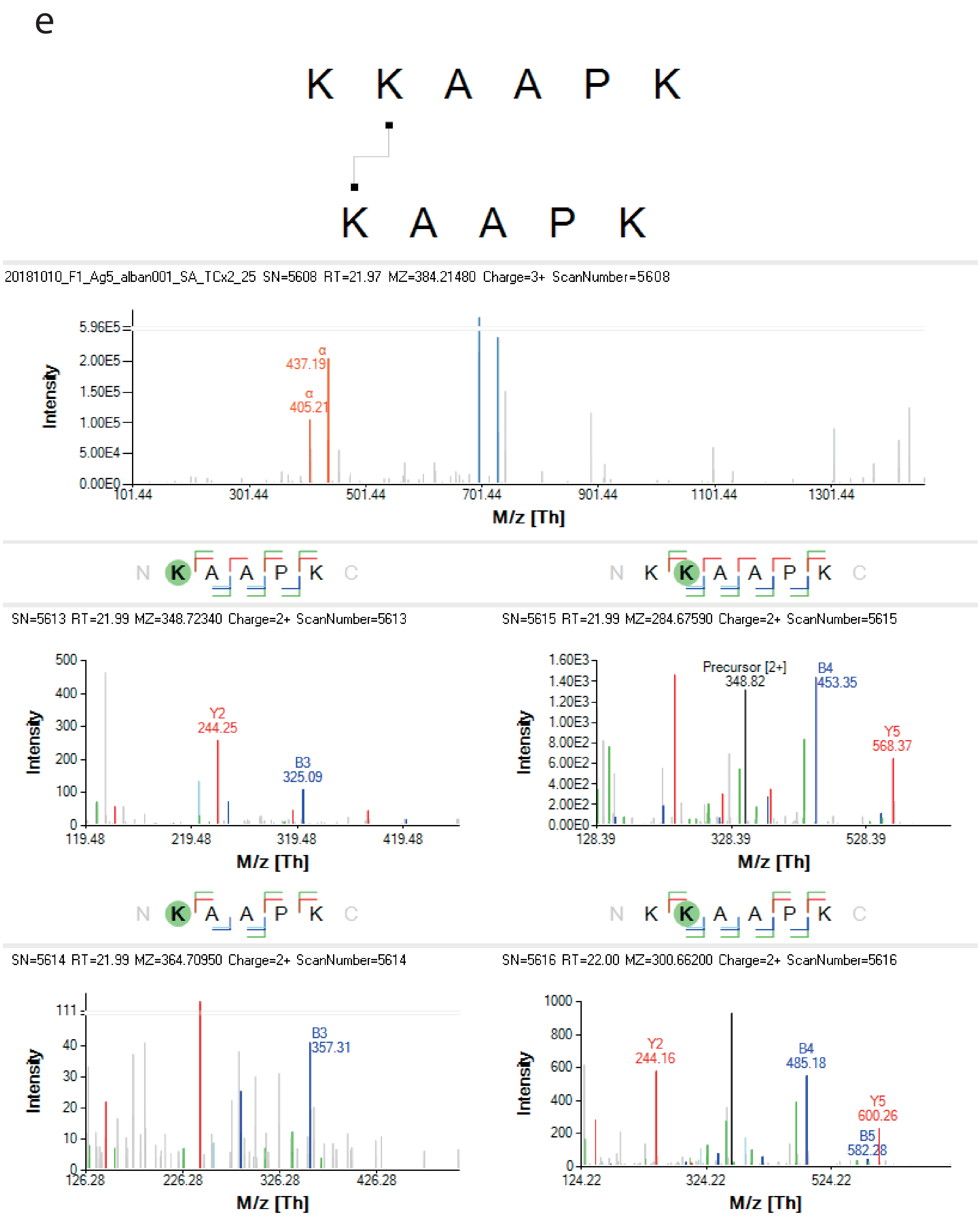

Supplementary Figure 11. MS/MS spectra of the DSSO self-links detected in thylakoid membranes for Lhcb1 (a-c) and Lhcb4.2 (d-f). Neighboring lysine residues are considered self-links when distance is below 7 Å. When MS2 spectra were not used for identification, the MS3 fragment spectra are shown.

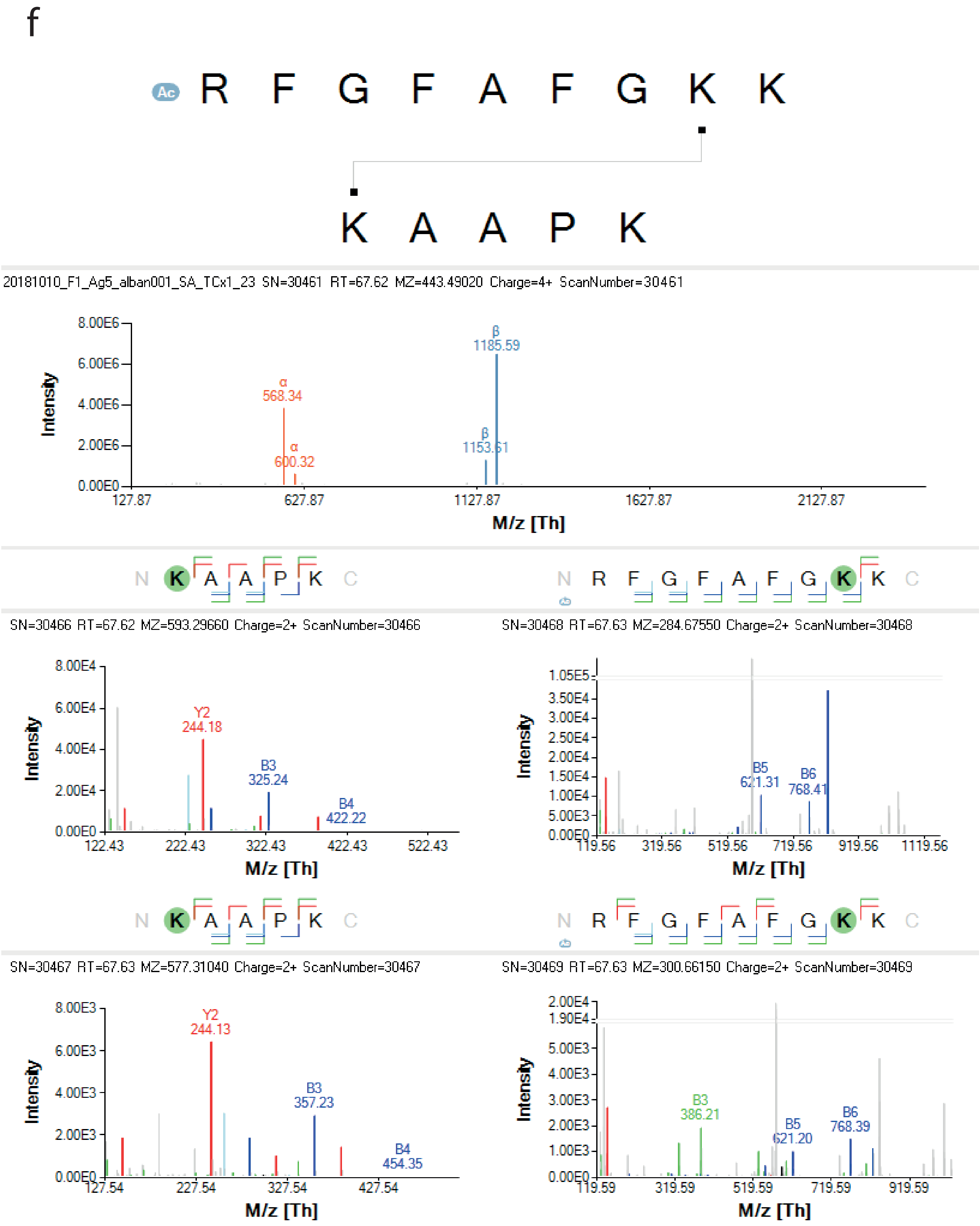

Supplementary Figure 11. MS/MS spectra of the DSSO self-links detected in thylakoid membranes for Lhcb1 (a-c) and Lhcb4.2 (d-f). Neighboring lysine residues are considered self-links when distance is below 7 Å. When MS2 spectra were not used for identification, the MS3 fragment spectra are shown.

Supplementary Note 1

Quantification of Lhcb1/Lhcb2 with labelled synthetic peptides

Selection and synthesis of internal peptide standards. To further corroborate our stoichiometry esti-mates obtained by TD-MS for the highly homologous Lhcb isoforms we conducted an additional absolute quantitation experiment. From previous bottom-up MS data[1,2], we could identify unique peptides for each of the primary isoforms Lhcb1_0081729 (IAGGPLGEVVDPLYPGGSFDPLGLADDPEAFAELK) and Lhcb2_0062077 (VGGGPLGEGLDPLYPGGAFDPLGLADDPDSFAELK), for both N-terminal truncated or complete forms (see Fig. 2d and Supplementary Data 2). These peptides were of sufficient length for detection and identification and did not contain Cysteine or Methionine residues, which eliminated possible adverse effects of substoichiometric modifications, e.g. Carbamidomethylation and/or Oxidation. The selected peptides were synthesized in-house and quantified by a BCA assay[3].

Peptide dimethyl-labelling and MS acquisition. The labelling of both the synthetic and endogenous peptides was performed in parallel at the peptide level according to our established and widely used quantitative on-column dimethyl-labelling protocol[4]. The heavy-labelled ($^{13}CHD_2$) synthetic peptides were consequently spiked into the light-labelled (CH_3) tryptic digest of the PSII-LHCIIsc sample, isolated from plants grown at moderate light intensity (*i.e.* C sample) at a final concentration of 100 nM. Two biological replicates were injected and separated on a 50 cm × 75 µm C_{18} analytical column, packed in-house (Poroshell 120 EC- C_{18} / 2.7 µm) connected to Agilent 1290 LC system and an Orbitrap Fusion Tribrid Mass Spectrometer (Thermo Fisher Scientific, Bremen, Germany). First, two survey MS runs of 60 min were performed with standard acquisition settings in order to generate an inclusion list of the mono-isotopic masses and retention times for the selected peptide precursors for subsequent quantitative targeted acquisition. The MS acquisition settings were specified as follows:

**scan event MS1**	Scan Range (m/z) = 375-2000
	Maximum Injection Time (ms) = 50
	AGC Target = 400000
	Normalized AGC Target = 100%
	RF Lens (%) = 60
**Filter Charge State**	Include charge state(s) = 2-6
	Include undetermined charge states = True
**Filter Dynamic Exclusion**	Exclude after n times = 1
	Exclusion duration (s) = 8
	Mass Tolerance = ppm
	Mass tolerance low = 10
	Mass tolerance high = 10
**Filter Targeted Inclusion Mass List**	Mass tolerance low = 25 ppm
	Mass tolerance high = 25 ppm
	Mass list type = m/z & z
	Ignore charge state requirement for unassigned ions = True

>>>>>>>>**Mass List Table**<<<<<<<	
m/z	z
640.1386	5
720.5887	5
799.9214	4
882.9644	4
900.484	4
912.8358	3
1066.2261	3
1176.9502	3
1293.6698	3
1764.9216	2
1799.9607	2
1060.8632	3
1171.5872	3
1194.9467	3
>>>>>>>>**END Mass List Table**<<<<<<<	

**scan event MS2 (CID)**	Isolation Mode = Quadrupole
	Isolation Window = 3
	ActivationType = CID
	Collision Energy (%) = 35
	Activation Time (ms) = 10
	Orbitrap Resolution = 30K
	Maximum Injection Time (ms) = 54
	AGC Target = 50000
	Normalized AGC Target = 100%
**scan event MS2 (HCD)**	Isolation Mode = Quadrupole
	Isolation Window = 3
	ActivationType = HCD
	Collision Energy Mode = Fixed
	Collision Energy (%) = 35
	Orbitrap Resolution = 30K
	Maximum Injection Time (ms) = 54
	AGC Target = 50000

Data processing and main results. The recorded raw-files were processed with MaxQuant (v1.6.7.0) with default settings[5]. The only alteration was the specification of dimethyl-labelling-based quantification with the following labels, denoted as light: DimethLys0 & DimethNterm0 and heavy: DimethLys8 & DimethNterm8. For the database search, we used the sequences of the proteoforms obtained with the top-down MS experiment and provided in Supplementary Data 2.
The results show an Lhcb1:Lhcb2 ratio of ~3.2:1 (Supplementary Figure 4), which is comparable with the intensity-based ratio inferred from the TD-MS measurements (ratio of ~2.9:1 shown in Figure 2c in the main manuscript). We performed this absolute quantitation experiment on the control light (C) PSII-LHCIIsc sample, where the most abundant supercomplex is of type C_2S_2M. Considering the most probable configuration for the LHCII M trimer as Lhcb1:Lhcb3 in a ratio 2:1 and a configuration for the S trimer with Lhcb1:Lhcb2 in a ratio 2:1, then in the C_2S_2M supercomplex we expect a composition of 6 Lhcb1 and 2 Lhcb2, which leads to a Lhcb1:Lhcb2 ratio of 3:1. In conclusion, the experimentally measured ratio for Lhcb1-Lhcb2 confirms the heterotrimeric composition of the S-trimer with Lhcb1: Lhcb2 in a ratio 2:1.

Data availability. All MS raw-files and MaxQuant results are deposited to the ProteomeXchange Consortium (http://proteomecentral.proteomexchange.org) via the PRIDE partner repository[6] with the dataset identifier PXD017382.

Supplementary References

1. Albanese, P. *et al.* Dynamic reorganization of photosystem II supercomplexes in response to variations in light intensities. B*iochim. Biophys. Acta - Bioenerg.* 1857, 1651–1660 (2016).

2. Albanese, P. *et al.* Thylakoid proteome modulation in pea plants grown at different irradiances: quantitative proteomic profiling in a non-model organism aided by transcriptomic data integration. *Plant J.* 96, 786–800 (2018).

3. Smith, P. K. *et al.* Measurement of protein using bicinchoninic acid. *Anal. Biochem.* 150, 76–85 (1985).

4. Boersema, P. J., Raijmakers, R., Lemeer, S., Mohammed, S. & Heck, A. J. R. Multiplex peptide stable isotope dimethyl labeling for quantitative proteomics. *Nat. Protoc.* 4, 484–494 (2009).

5. Cox, J. & Mann, M. MaxQuant enables high peptide identification rates, individualized p.p.b.-range mass accuracies and proteome-wide protein quantification. *Nat. Biotechnol.* 26, 1367–1372 (2008).

6. Vizcaíno, J. A. *et al.* The Proteomics Identifications (PRIDE) database and associated tools: Status in 2013. *Nucleic Acids Res.* 41, D1063–D1069 (2013).
