# Supplementary material for: How paired PSII–LHCII supercomplexes mediate the stacking of plant thylakoid membranes unveiled by structural mass-spectrometry
Source: Nat Commun. 2020 Mar 13;11:1361. doi: 10.1038/s41467-020-15184-1 (PMC7069969; doi:10.1038/s41467-020-15184-1)
Supplement: Supplementary file 2 — Description of Additional Supplementary Files [file 41467_2020_15184_MOESM2_ESM.pdf]

Description of Additional Supplementary Files

Supplementary Data 1. List of proteins (**a**) and their proteoforms (**b**) detected by TD-MS. (**a**) Homology identification and quantification of all proteins. (**b**) Quantification of proteoforms with acetylation on the N-terminal domain, if present, indicated as the length of the first b-ion.

Supplementary Data 2. Complete protein databases used for the search in FASTA format.

Supplementary Data 3. List of identified crosslinked peptide pairs (sub-tables 1,3,5,7,9,11), and their crosslink-spectrum matches (sub-tables 2,4,6,8,10,12), of PSII-LHCIIsc isolated in L, C, or H light conditions and treated with either DSSO (sub-tables 1 to 6 and 13) or EDC (sub-tables 7 to 12). Sub-table 13 is a list of crosslinked peptide pairs identified searching C_DSSO raw files against the "PSII-LHCII_transcriptome_UniprotKB" FASTA database (Supplementary Data 2).

Supplementary Data 4. Complete set of predicted structures of LHCII and PSII subunits, and all nine theoretical models (named model 1-1, 1-2, 1-3, 2-1, 2-2, 2-3, 3-1, 3-2, 3-3) used for scoring Lhcb2 position, in PDB format.

Supplementary Data 5. Scoring the position of Lhcb2 within the S-trimer in the nine theoretical structural models of PSII-LHCIIsc. (**a**) List of inter-protein crosslinks of Lhcb2 used to score its positioning. (**b**) Scoring of each theoretical model (see Supplementary Data 4 for PDB structures) based on the occurrence of the interlinks in (**a**) within the distance cut-off. (**c**) Total number of crosslinks within the distance cut-off for each model.

Supplementary Data 6. List of identified crosslinked peptide pairs (sub-tables 1,3,5), and their crosslink-spectrum matches (sub-tables 2,4,6) on the three replicates (rep1-3) of thylakoid membranes isolated from plants grown under moderate light (C).
